# Consumer understanding of climate targets is low and hard to improve: evidence from three online studies

**DOI:** 10.1038/s41598-026-52744-9

**Published:** 2026-09-11

**Authors:** Vittoria Battocletti, Alfredo Desiato, Alessandro Romano, Chiara Sotis, Tobias H. Tröger

**Affiliations:** 1 University of Amsterdam, Amsterdam, Netherlands; 2 Aston University, Birmingham, UK; 3 Bocconi University, Milan, Italy; 4 Northwestern University, Evanston, USA; 5 Goethe University, Leibniz Institute SAFE and ECGI, Frankfurt, Germany

**Keywords:** Environmental social sciences, Psychology, Psychology

## Abstract

Many firms publicize net-zero or carbon neutral targets, yet it is unclear whether consumers understand these targets. Policy proposals–as well as recent court rulings–call on firms to explain what those targets mean. We test whether such explanations enhance consumer understanding in two online studies with nationally representative U.S. samples (N=300; N=1,500) and in a large-scale webcam-based eye-tracking experiment (N=500). Baseline knowledge is low: 95 percent of respondents fail to name even one defining feature of net-zero or carbon neutral targets, yet they are willing to pay a price premium for gift cards from companies with such targets. Concise disclosures raise objective understanding when a single product is shown, but none of the formats alter willingness to pay. In a more realistic setting with multiple products and competing cues, disclosures attract substantial gaze time, yet no longer improve comprehension; the visually salient color-coded format is associated with more misconceptions. These findings contribute to the growing evidence that individual-level measures, such as information provision, may be a less effective response to systemic problems like global warming than previously thought.

## Introduction

The push to limit global warming to $1.5^{\circ }$ C has prompted thousands of firms to announce climate targets. More than 3,000 corporations have already validated net-zero targets through the Science Based Targets initiative (SBTi), and the number of consumer-facing products marketed as carbon neutral grows daily.

Key actors such as the SBTi, the International Organization for Standardization (ISO), and the United Nations (UN) agree on two fundamental distinctions between carbon neutral and net-zero targets. Net-zero requires deep, science-aligned cuts across all greenhouse-gas (GHG) emissions, with removals used only for the unavoidable residual. To become carbon neutral, by contrast, a firm can rely entirely on offsets and typically concerns $CO_2$ alone.

However, recent evidence shows that consumers rarely understand the meaning of carbon neutral and net-zero targets, let alone the differences between them. Fewer than one in ten U.S. consumers can accurately describe what either target entails^1^. Surveys in the U.K. and Europe report similar results^2,3^. Even though consumers do not understand the meaning of these targets, evidence suggests that they are willing to pay a premium for products or brands associated with them^4–7^. As a result, firms may exploit consumers’ limited understanding of climate targets by advertising targets that – while technically accurate – sound more ambitious than they actually are.

Regulators and courts on both sides of the Atlantic are wrestling with how to address the information asymmetry between firms communicating climate targets and the consumers receiving them. Among the measures most often discussed are (*i*) banning potentially confusing climate targets –as in the proposed EU Green Claims Directive– and (*ii*) requiring firms to disclose information explaining their climate targets. This raises a fundamental question for policy-makers and behavioral scientists alike: can mandated climate-target disclosures bridge the gap between corporate rhetoric and informed consumer choice?

Whether such information-centric interventions–an archetypal “individual-frame” (i-frame) policy tool–actually attract consumers’ visual attention, improve unprompted consumer understanding of climate targets, and shift willingness to pay (WTP) in realistic shopping environments remains unknown. Previous evidence on disclosure efficacy is mixed and largely confined to financial products^8,9^ or energy-use labels^10,11^, leaving the climate-target context underexplored.

We address this gap through three studies that combine online surveys with a large-scale, webcam-based eye tracking experiment. In study I, we measure baseline knowledge of climate-target meaning in a nationally representative U.S. sample (N = 300). In study II, we randomize a nationally representative U.S. sample of 1,500 respondents who receive either no information regarding climate targets or one of three disclosure formats–information, bullet points, or color-coded information. We elicit both objective understanding of climate targets and incentive-compatible WTP for gift cards linked to such targets. In study III, we embed the two concise formats in a simulated e-commerce setting and record eye movements from 500 participants to analyze how attention mediates comprehension when multiple product cues compete for notice.

Across our studies, information provision improves only visual recognition–performance on multiple-choice questions about the features of a given target– when a single product is shown. It has little effect on unprompted understanding, tested with an open-ended question that asks participants to state the key differences between net-zero and carbon neutral targets. Furthermore, it leaves WTP unchanged. In a setting where multiple cues compete for attention, any comprehension gain disappears; the visually salient traffic lights label even amplifies misconceptions. Eye tracking data reveal that participants devote substantial gaze time to the disclosures, indicating that the failure lies not in visibility but in processing and interpretation. These findings question the effectiveness of i-frame information remedies to improve consumers’ understanding of climate targets.

## Results

### Study I: respondents’ knowledge about climate targets

Consistent with earlier studies, Study I confirms that people have limited knowledge of the most common climate targets^1,2^. In a representative sample of 300 U.S residents, only 5.25% of participants correctly identified one of the differences between net-zero and carbon neutral targets (mean=0.053, SD=0.223), and no participant identified both differences.

### Study II

**Fig. 1:**
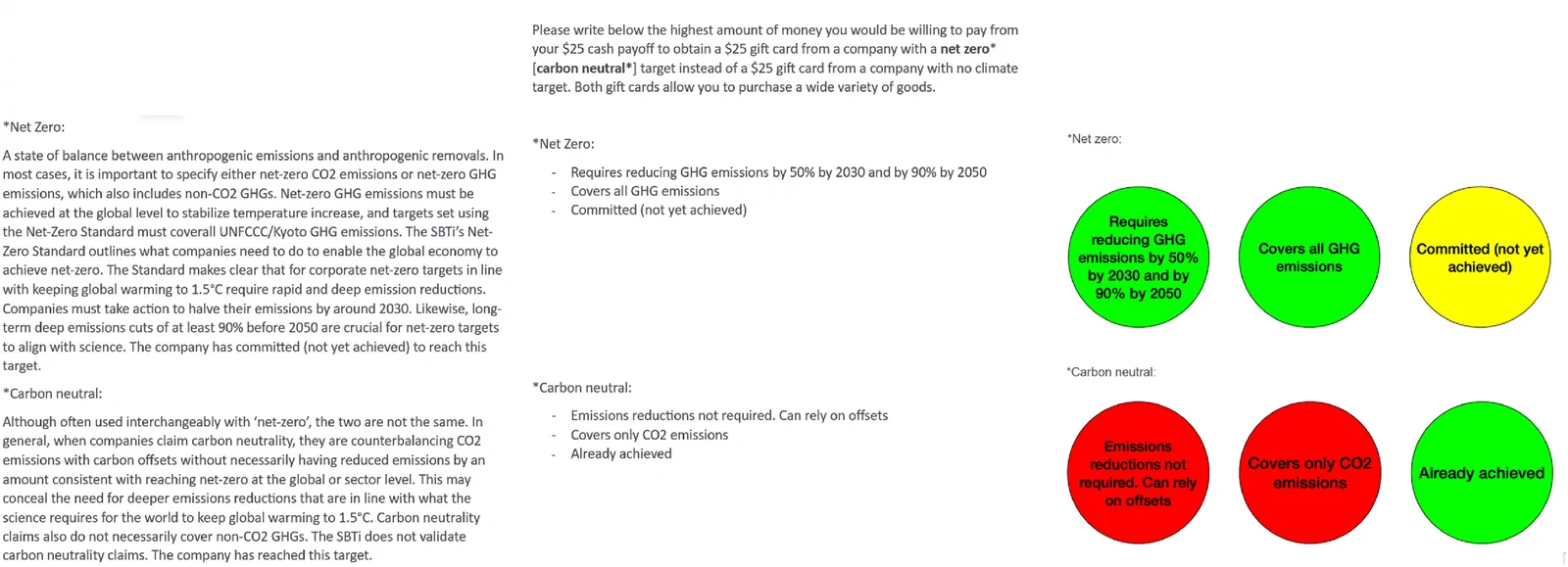
Information provided to participants regarding net-zero and carbon neutral as displayed in the three conditions: information (left), bullet points (center), and traffic lights (right).

In Study II, we recruit a representative sample of 1,500 U.S. residents to test how information provision affects (i) respondents’ understanding of a firm’s climate target and (ii) their WTP for a gift card from that firm. We randomly assign participants to one of four disclosure groups: no disclosure (control), information (full definition), bullet points, and traffic lights. Figure 1 shows the disclosure formats presented to the treatment groups.

We measure total understanding on a 0 – 10 scale. We assign participants 1 point for each correct answer; up to 2 points for the open-ended question (“unprompted”) and up to 8 points from eight multiple-choice (“visual recognition”) items (see full rubric in the Methods).

We elicit the WTP with an incentive-compatible Becker–DeGroot–Marschak (BDM) procedure: each respondent states how much of a potential $25 cash bonus they would forgo to switch from a gift card issued by a firm without a climate target to one from a firm with either a net-zero or a carbon neutral target (shown in a randomized order).

We analyse two dimensions: (i) *within-subjects* contrasts, comparing participants’ responses to net-zero versus carbon neutral targets presented in the same format, and (ii) *between-subjects* contrasts, comparing responses across formats for the same target.

For within-subjects contrasts between net-zero and carbon-neutral targets, we report p-values and t-statistics from standard two-sided paired t-tests. For between-subjects contrasts, we report the results of ordered logit regressions that interact treatment groups with each level of the mechanisms, controlling for respondents’ consistency and demographic characteristics using robust standard errors. In both cases, we report the p-value from two-sided tests.

To investigate whether the impact of the mechanisms varies by level, we run regressions interacting each level of the mechanism with the treatment groups (Table 1).

#### Understanding

**Table 1:** Summary statistics by group and total (mean and standard deviation) for understanding variables in Study II.

	Group				
	All	Control	Information	Bullet points	Traffic lights
Unprompted understanding	0.157 (0.391)	0.070 (0.256)	0.236 (0.468)	0.229 (0.457)	0.175 (0.407)
Visual recognition	3.948 (1.977)	3.019 (1.460)	3.734 (1.836)	4.781 (2.061)	4.239 (2.049)
Total understanding	4.126 (2.112)	3.089 (1.491)	3.970 (1.981)	5.011 (2.207)	4.414 (2.203)

Focusing on unprompted understanding, we find that all treatment groups have a significant effect compared to the control group (bullet points *p<0.001*; information *p=0.004*; traffic lights *p=0.002*). We observe no statistically significant difference among the treatment groups when testing them through pairwise comparisons and including our set of controls.

With respect to the multiple-choice questions (“visual recognition”), all three treatment groups outperform the control group, with bullet points producing the largest effect (bullet points: *p<0.001*; information: *p=0.008*; traffic lights: *p=0.001*).

Further, we test whether concise formats reduce outright misconceptions. We study whether respondents in the bullet points and traffic lights conditions are less likely to supply statements that contradict the disclosure. We find no significant differences among groups.

**Fig. 2:**
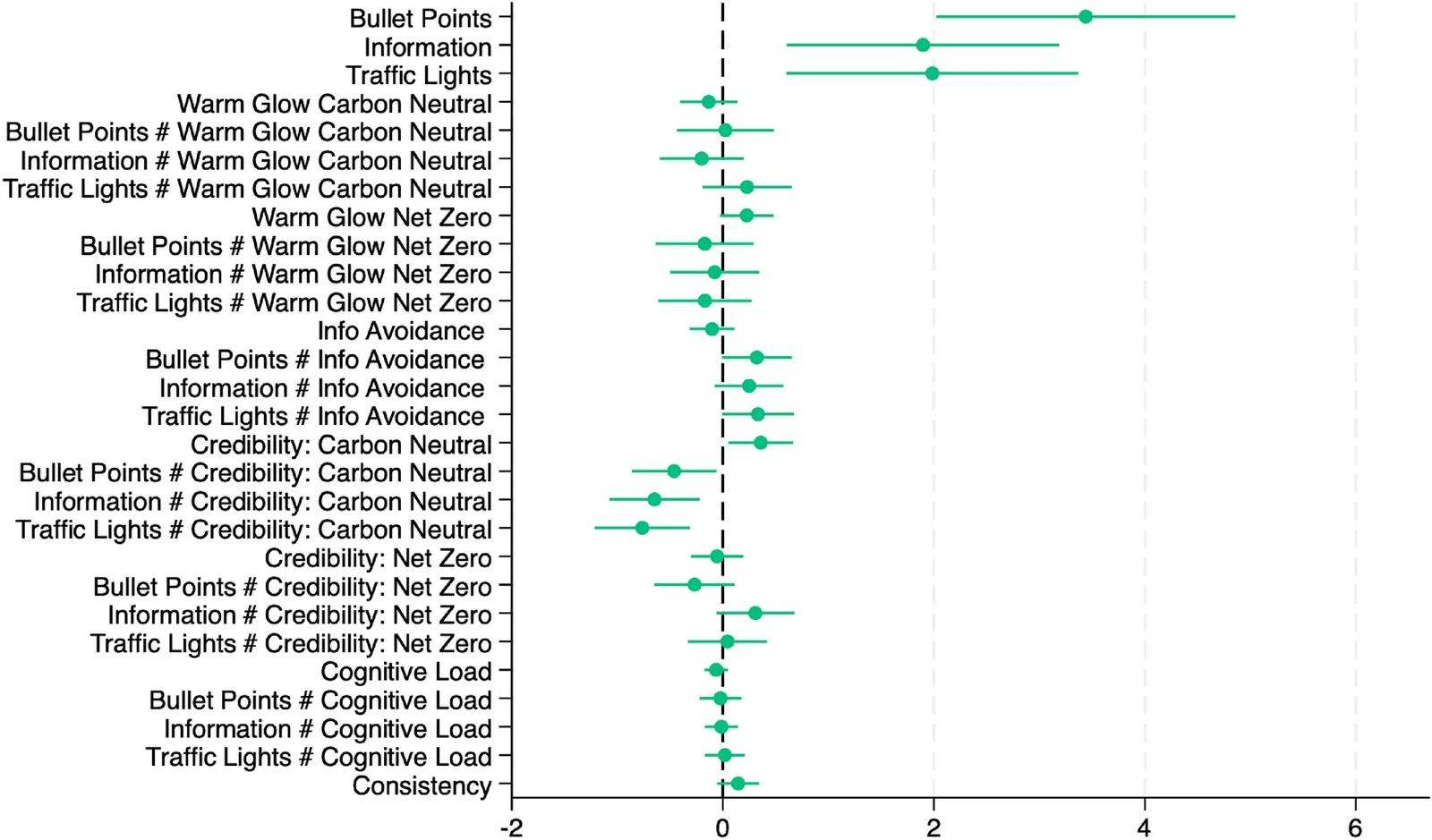
Drivers of understanding. Odds ratios (dots) and 95% confidence intervals from an ordered-logit model of total understanding, with robust standard errors. Predictors include disclosure group, four mechanisms (warm glow, information avoidance, credibility, cognitive load), their interactions with group, and participants’ choice consistency and demographics.

To explore mechanisms, we inspect the interaction coefficients from the ordered-logit model. Apart from the treatment indicators themselves, the perceived credibility of carbon neutrality is the only mechanism that predicts total understanding (Fig.2): credibility of carbon neutrality is positively associated with understanding in the control group and is negatively associated in each treatment group relative to control. Information avoidance in all treatment groups increases understanding, but is only marginally significant.

#### Willingness to pay

Next, we examine respondents’ WTP for gift cards issued by firms with a carbon neutral, a net-zero, or no climate target.

Participants exhibit a substantial WTP for both net-zero and carbon neutral targets (see Table 2). In the full sample, respondents are willing to pay an average of $6.49 (SD=6.11) – about 26% of the gift card’s face value – to switch from a gift card issued by a firm with no climate target to one with a carbon neutral target and $7.15 (SD= 6.35) – roughly 29% of the gift card’s face value – for a net-zero target.

We acknowledge that this premium may partly reflect demand effects and the fact that participants are asked to forego windfall money rather than pay out of pocket. However, the goal of the paper is not to provide a precise estimate of WTP for climate targets. Instead, we first seek to replicate the well-established finding that WTP for green attributes is positive; this is intuitive, since otherwise firms would have no incentive to set and communicate climate targets. Second, once we demonstrate that such a premium exists while understanding remains low, we can identify, within our setting, conditions under which consumers may reward claims that they perceive as more ambitious than they actually are, thereby creating incentives for firms to make such claims. Third, our main focus is on relative WTP across disclosure formats. It is unlikely that demand effects or the use of windfall money affect conditions differentially. Therefore, we can still isolate the treatment effect.

**Table 2:** Mean and standard deviation for the questions used to assess the participants’ WTP by group in Study II.

	Group									
	All		Control		Information		Bullet points		Traffic lights	
	mean	sd	mean	sd	mean	sd	mean	sd	mean	sd
WTP carbon neutral	6.49	6.11	7.03	6.19	6.30	6.12	6.19	5.72	6.46	6.41
WTP net zero	7.15	6.35	7.18	6.31	7.09	6.07	6.57	5.87	7.75	7.05
WTP Difference	0.65	3.99	0.15	3.87	0.79	3.41	0.38	4.37	1.30	4.15
Observations	1487		370		365		375		377	

In the control condition, the difference in the WTP between carbon neutral and net-zero targets is only $0.15 (SD= 3.86, p=0.46), consistent with most respondents being unaware of the differences between the two targets. This wedge increases in the treatment conditions: information ($0.79, SD=3.41, *p<0.001*), bullet points ($0.38, SD= 4.37, *p=0.096*), and traffic lights ($1.3, SD= 4.15, *p<0.001*). However, these differences are no longer significant when controlling for covariates (see supplementary materials).

**Fig. 3:**
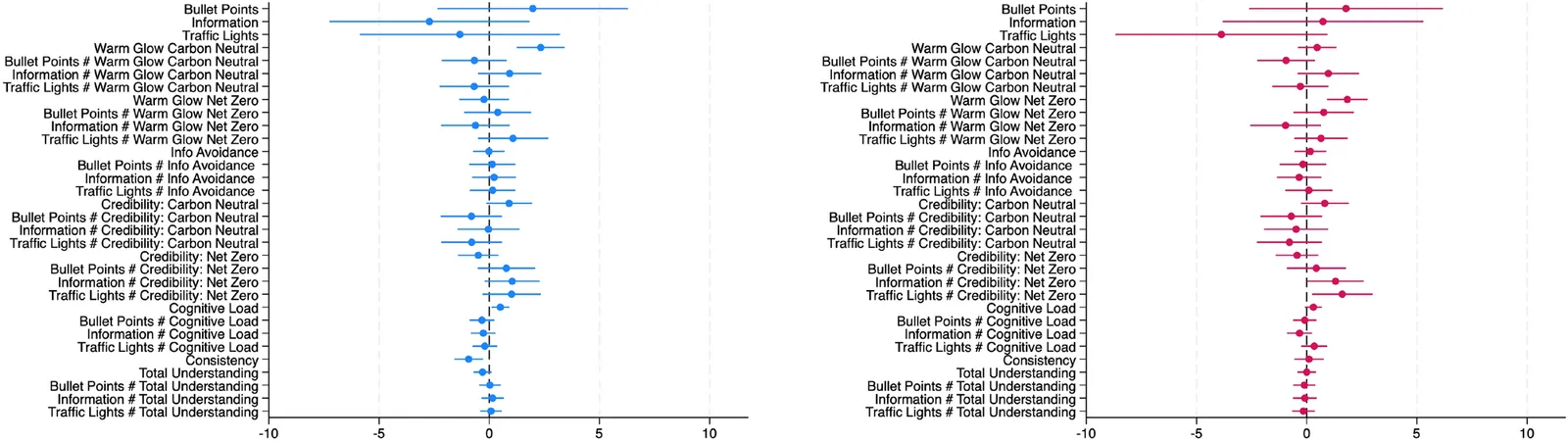
Drivers of WTP: coefficients and 95% confidence intervals from OLS regressions with robust standard errors, using WTP for net-zero (left panel) and carbon neutral (right panel) as the dependent variable. The regressions control for treatment group, each mechanism, its interaction with the treatment, participants’ choice consistency, and demographics.

In fact, the WTP for a climate target depends only on the warm glow that a respondent feels towards it (Fig. 3), which strictly increases the WTP to pay for that target in the control group. Respondents pay more when they experience a stronger warm glow, whereas better understanding does not significantly predict WTP for either target. For the net zero gift card the WTP also increases when its credibility is perceived as higher, but only in the information and traffic lights groups. For the carbon neutral gift card the WTP increases in the control group when participants perceive a higher cognitive load.

We report the full regression tables in the supplementary materials.

To assess whether the WTP results are driven by participants stating a zero WTP, we conduct additional robustness checks. We test whether the share of respondents reporting zero WTP differs across treatment groups and find no statistically significant differences. We also report robustness checks excluding participants who failed the comprehension checks for the BDM procedure. These analyses are reported in the supplementary materials.

### Study III

**Fig. 4:**
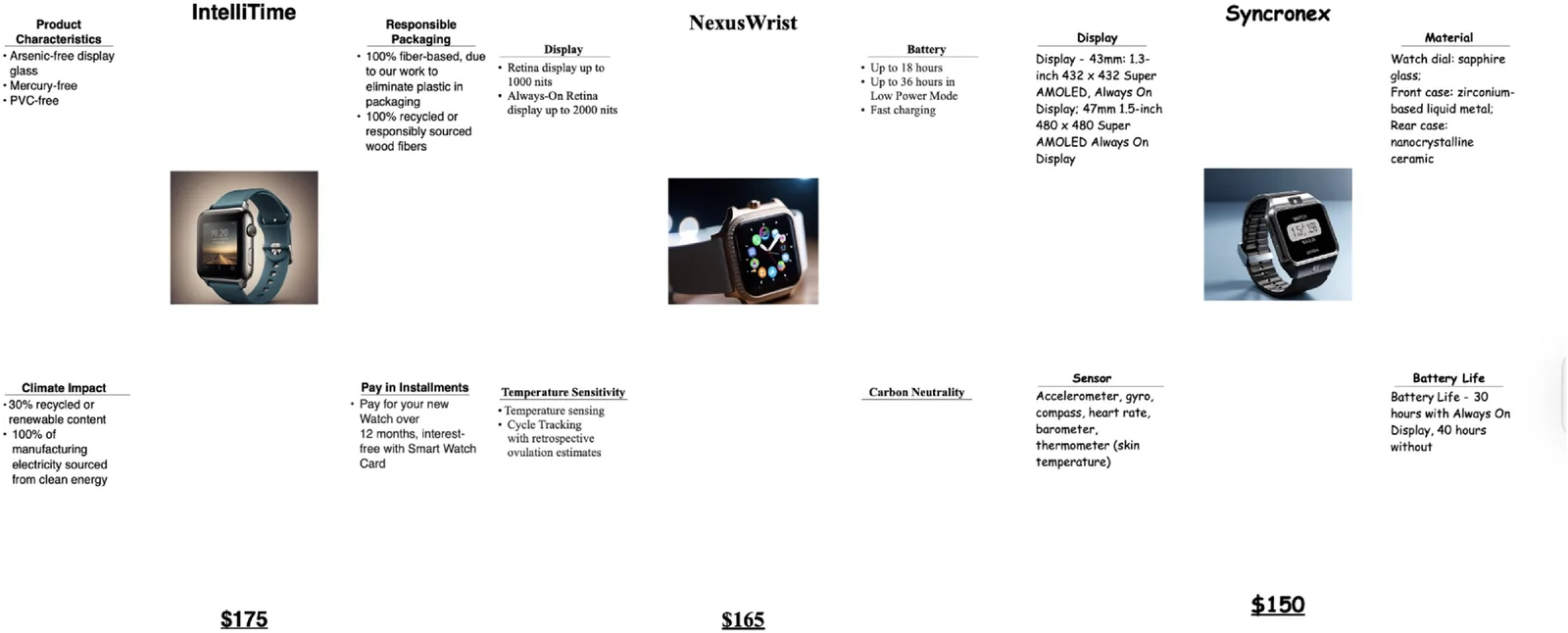
The visuals regarding the three smart watches designed for the third experiment and were built on the ones used by Apple for its Apple Watch Series 9. The treatments consisted of changes in the presentation of the smart watch NexusWrist (bottom right).

**Fig. 5:**
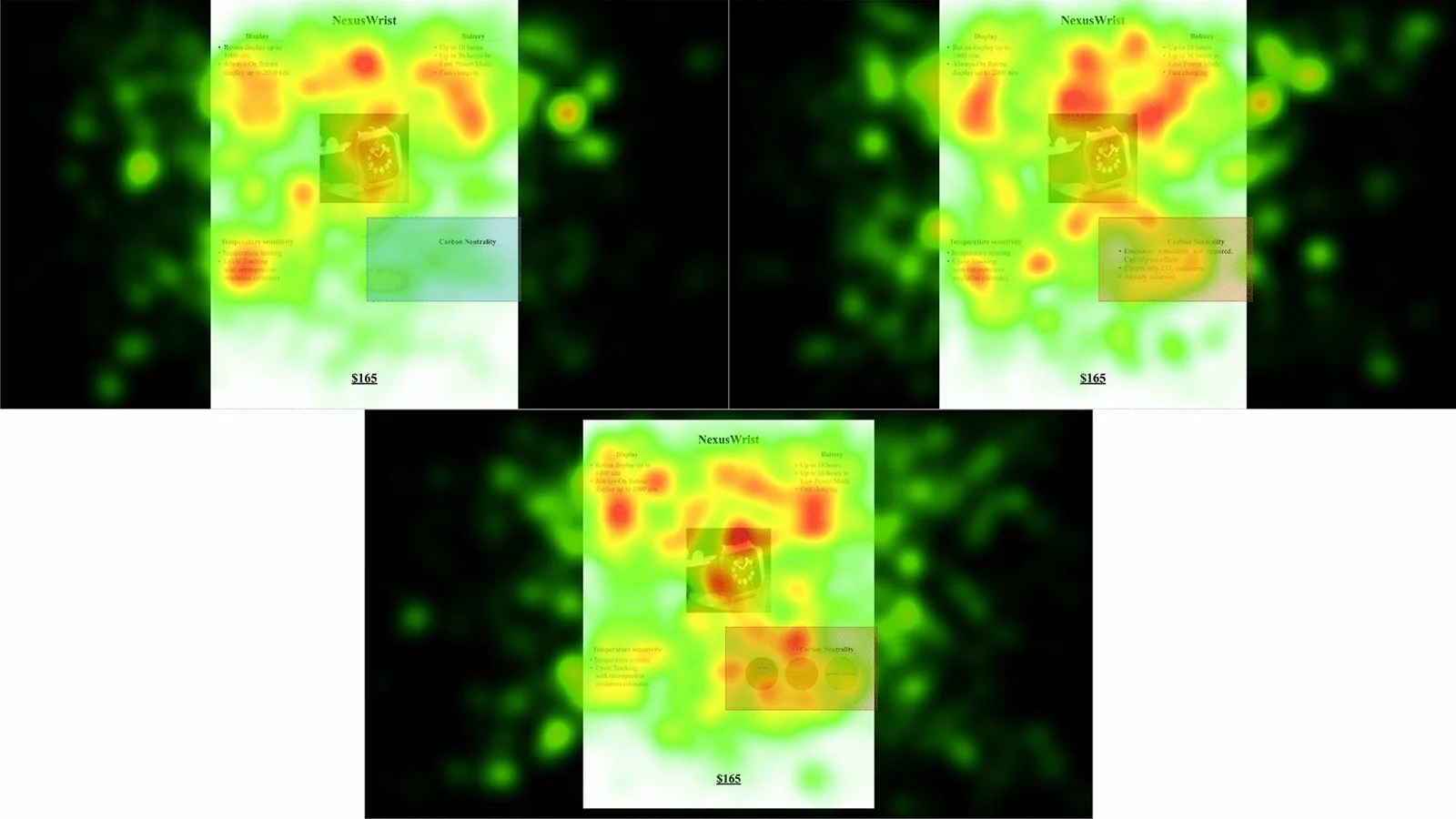
Heat-maps showing the AOI and respondents’ fixations distribution while looking at the treated picture in the **a)** control group (top-left), **b)** bullet points group (top-right), **c)** traffic lights group (bottom-center).

In study III, we recruit a sample of 500 U.S. residents to measure how much visual attention respondents devote to information related to climate targets and how well they understand it when they encounter multiple pieces of information across several products (Fig. 4). Our area of interest (AOI) is the section of the screen that displays the treatment (Fig. 5).

To test differences across the treatment groups, we rely on two-sample, two-sided t-tests using unequal variances (Welsch’s t-test) and report the associated t-statistic. In both cases, we report the p-value from two-sided tests.

#### Respondents’ attention when exposed to multiple pieces of information

We report the results for a sample that excludes only those recordings that did not meet the minimal acquisition criteria (data quality above 50% and accuracy of at most $10.0^{\circ }$). As a robustness check, we present in the supplementary materials the analysis including only participants whose eye-tracking data meet a higher threshold (data quality above 80% and accuracy of at most $3.0^{\circ }$).

**Table 3:** Summary statistics and t-tests for the Dwell Time(%), Dwell time (ms) and Time-To-First-Fixation (TTFF) in Study III. Row 1 reports the number of respondents who had no fixations in the AOI. For each measure, we report both values conditional on having at least one fixation in the AOI and unconditional values, which include all participants (assigning zero dwell time to those with no fixation). Means and standard deviations (in parentheses) are shown for each treatment group. Columns 5 and 6 report the differences between the bullet points (BP) group and the control group, and the traffic lights (TL) group and the control group. The p-values reported refer to two-sided t-tests with unequal variances.

	Control	Bullet points	Traffic lights	BP-control	TL-control
No fixation detected in the AOI	82	54	32	-28	-50
Dwell time (%)					
Conditional on fixation detected	9.08 (10.62)	19.23 (18.52)	21.16 (18.99)	10.15, p<0.0001	12.08, p<0.0001
Unconditional	4.57 (8.78)	11.92 (17.30)	16.88 (18.98)	7.35 , p<0.0001	12.31, p<0.0001
Dwell time (ms)					
Conditional on fixation detected	1576.28 (2185.84)	3318.20 (3172.62)	4111.08 (5067.21)	1741.93, p<0.0001	2534.80, p<0.0001
Unconditional	792.91 (1736.05)	2056.35 (2970.50)	3278.45 (4815.63)	1263.44, p<0.0001	2485.54, p<0.0001
TTFF	7407.85 (4969.92)	7803.32 (5707.00)	6417.81 (6059.13)	395.47, p=0.6290	-990.03, p=0.1985
N	165	142	158	307	323

Dwell time measures indicate that the traffic lights format attracts the most visual attention. Relative to both the control and bullet points groups, a larger share of participants fixate on the AOI, their dwell time is longer, and they spend a higher share of total viewing time on the treated area (see Table 3 and Fig. 5). The TTFF for traffic lights format is also shorter, but the difference is not statistically significant when we consider only participants with high eye tracking data quality. The bullet points format also draws attention, though less effectively than traffic lights. Compared with the control group, a greater proportion of bullet points participants fixate on the AOI, and they spend more time there overall, but their average TTFF is slightly longer.

#### Respondents’ understanding of climate targets when exposed to multiple pieces of information

We also explore participants’ understanding of climate targets in a setting that replicates a typical shopping experience, where several pieces of information relate to multiple products. As in Study I, no respondent identifies both defining features of the carbon neutral target.

This observation suggests that the comprehension gains from information provision disappear when additional product cues compete for attention. To test whether concise formats at least reduce outright misconceptions.

In line with Study I, we study whether concise formats reduce misconception. We find the opposite for the traffic light group: this format is actually associated with a higher rate of conflicting responses. Respondents in the traffic lights condition are more likely to incorrectly associate carbon neutrality with emission reductions, low emissions, or no emissions compared to the control group. The share of answers that conflict with the information provided is 18.5% (SD = 0.389) in the control group, 20.5% (SD = 0.405) in the bullet points group, and 26.4% (SD = 0.442) in the traffic lights group. The difference in means between the traffic lights and control groups is statistically significant (diff = 0.08, *p =0.085*), while the difference between the bullet points and control groups is not (diff = 0.021, *p=0.642*). There is also no difference between the treatment groups (diff = 0.059 , *p=0.228*). Logit regressions with robust standard errors–controlling for demographics and cognitive load–yield the same pattern (see supplementary materials).

## Discussion

We find that respondents exhibit both a positive WTP for climate targets and a very low understanding of what those targets actually mean. This suggests that firms may be able to charge significant premia by setting targets that are less ambitious than they sound. We test whether providing consumers with information on the core characteristics of climate targets improves understanding and enables more informed choices.

Studies II and III suggest that improving consumers’ understanding of climate targets may not be easy. In both studies, we observe that information provision does not improve respondents’ unprompted understanding of climate targets. Moreover, in a more realistic setting where multiple product cues compete for attention, information provision may even increase confusion. When recipients view several pieces of information, participants who see the traffic lights format–the one which draws the most visual attention–are more likely to claim that carbon neutral entails emissions reduction, the opposite of its actual definition provided in the treatment. A tentative explanation for these unexpected results relates to the standard finding that processing negation is effortful and cognitively demanding^12,13^. Therefore, the “no reductions” clause may not be encoded, or might be encoded with the NOT operator, but when people retrieve the meaning, the core term (“emission reductions”) remains more salient rather than the “no.” Similarly, when thinking about climate targets, people’s mental models may already link them to reducing emissions. That expectation means “emission reductions” is present in the situation model. The negation (“no”) makes it harder to suppress that default association^14^.

These findings are consistent with the idea that information on complex issues–especially when consumers are already flooded with cues–may at best enhance shallow forms of understanding, such as visual recognition, yet is unlikely to improve unprompted understanding. Thus, in realistic settings, information provision may not only fail to inform but also increase confusion and impose a cost by diverting attention to disclosures that consumers ultimately do not understand.

In the Results, we show that information provision does not alter WTP and that WTP is not correlated with understanding. This lack of correlation is consistent with prior work suggesting that both consumers and investors are largely indifferent to the specific environmental effects of the products they purchase. Instead, they are more interested in the “expressive value” of the purchase, which is the utility derived from expressing one’s identity through consumer choices^9,15–17^. According to this literature, people’s WTP is more closely connected to the expressed support for an issue and anticipated moral satisfaction from contributing than to the impact of the contribution on the issue^18^.

Our results relate to the debate on systemic frame (s-frame) interventions and individual frame (i-frame) interventions. Although social scientists have often emphasized how i-frame interventions can play a significant role in achieving social goals^19,20^, recent evidence has suggested that i-frame interventions have a smaller impact than usually assumed^21–23^. Our findings further reinforce the idea that for a systemic challenge like climate change, an i-frame solution such as information provision may be less effective than previously thought and might backfire^24,25^.

A possible counter-argument is that comprehension may improve with repeated exposure. Participants in our study see the disclosure only once. If regulators mandated such disclosures, repeated exposure could foster understanding. As our design does not capture such dynamics, future work should examine the long-term effects of information provision.

## Methods

We conduct three studies cleared by the ethical committees of Bocconi University, Goethe University, and the London School of Economics. All study participants provided written informed consent. All methods were carried out in accordance with relevant guidelines and regulations. In Study I, we assess respondents’ knowledge regarding carbon neutral and net-zero climate targets. In Study II we investigate whether providing respondents with key information related to climate targets improves their understanding of such targets and influences their WTP for gift cards from firms setting such targets. In Study III, we test whether information provision improves understanding when participants are exposed to multiple product-related information. In addition, we test whether information that attracts more attention also enhances participants’ understanding.

### Study I: baseline understanding

For study I, we recruit a representative sample of *n=300* U.S. residents on Prolific.co. All participants are paid $0.67 for taking part in the experiment, equivalent to $8.07 per hour. In this study, participants answer an open-ended question in which they are asked to describe the two main differences between net-zero and carbon neutral. The two most important differences are: *i*) net-zero requires deep emission reductions, whereas carbon neutrality does not (“emission reductions”); *ii*) net-zero covers all greenhouse gases, whereas carbon neutrality focuses on $$CO_2$$ emissions (“all GHGs”).

We ask two independent coders, blind to the hypotheses, to assign two points to respondents who correctly identify both differences, one point to those who identify only one difference, and zero points to those who do not mention either difference. We only consider an answer correct when both coders code it as correct. We also collect demographic information about the participants. We further ask other questions that we do not include in the main analysis. The results are consistent with the finding that people have a poor understanding of climate targets.

### Study II: WTP for targets and information provision

For study II, we recruit a representative sample of *n=1500* U.S. residents on Prolific.co. All participants are paid $1.34 for taking part in the experiment, equivalent to $8.04 per hour. The full protocol of Study II is included in the supplementary materials.

At the beginning of the experiment, all respondents see the same text, explaining that many firms are setting climate targets like carbon neutral and net-zero, and that we are interested in knowing how much the respondents value these targets. We then explain that by taking part in the experiment, respondents will automatically enter a lottery in which eight participants will win a $25 gift card from a company with no climate target or $25 in cash via Prolific.

Participants are informed that they would be asked two questions to state how much of the $25 cash they might win, they would be willing to pay in order to switch from the gift card from a company without a climate target to: *i*) the gift card from a company with a net-zero target, *ii*) the gift card from a company with a carbon neutral target. The order of these two questions is randomized. To ensure that respondents understand the mechanism, we include two comprehension checks. If they select the wrong answer, we explain how to answer correctly; if they select the correct one, we inform them that their answer is correct.

To make the study incentive-compatible and avoid deception, we selected three companies that, at the time of the study’s launch, represented the three distinct target statuses: Apple had achieved a carbon neutral target, Walmart had pledged a net-zero target (which was subsequently removed by the SBTi), and Macy’s had no target.

Our framework is based on the Becker-DeGroot-Marschak^26^ mechanism as implemented in Kahneman, Knetsch and Thaler ^27^. The BDM has the important advantage of being incentive compatible^28^ and hence induces truthful valuations. To aid participants’ understanding of the mechanism and test their comprehension, we ask two questions about the procedure used to elicit the WTP and provide guidance on how to answer them.

Respondents are then randomly assigned to one of four groups: (i) control, (ii) information, (iii) bullet points and (iv) traffic lights. Participants are asked about their WTP for the gift card from firms with a climate target. The targets cannot be objectively ranked, as net-zero is a more comprehensive goal than carbon neutral, and the latter relies on carbon offsets that not always correspond to real emission reductions^29–31^. However, net-zero remains a mere promise, whereas the carbon neutral company has already achieved its goal. For this reason, we do not investigate whether consumers pay more for the “better” target. We only investigate if participants are willing to pay a premium for these targets, and whether information provision affects the size of such a premium.

Study 2 does not establish a normative ranking between the two targets.

Immediately after eliciting the WTP, we measure participants’ warm glow following the elicitation method used in Nunes & Schokkaert^32^ and respondents’ cognitive load following Paas & Van Merriënboer^33^.

We then test respondents’ understanding of the climate targets in two ways, aimed at measuring different facets of understanding. To measure unprompted understanding, we use the same question asked in Study I. In line with Study I, the coding was carried out by two coders to whom we provided a set of instructions to ensure unbiased coding (included in the supplementary materials). As in Study I, we asked the coders to assign one point for each difference between net-zero and carbon neutral that a respondent identifies correctly. Thus, the unprompted understanding score ranges between 0 and 2.

To measure a component of understanding that we label as visual recognition, we ask eight multiple-choice questions in which participants assign a given feature to either carbon neutral, net-zero, or neither of the targets. We assign one point for each characteristic that participants assign correctly. For instance, participants who assign “Emissions’ cut required” to net-zero receive one point. Hence, the score on the multiple-choice questions ranges between 0 and 8. We use this score to measure respondents’ visual recognition because answering these questions correctly requires recognizing information to which participants have been exposed on earlier screens.

Afterwards, we ask respondents a series of questions on their perception of climate targets and climate change. Specifically, we ask three questions modeled after those related to the avoidance of medical information, as we see strong similarities to the avoidance of environmental information. Lastly, we ask standard demographic questions and whether respondents have ever been informed or become aware that they are color-blind.

### Study III: eye tracking

Eye tracking provides insights into how people think, enabling researchers to understand what captures their attention^34^. Although eye tracking has been widely used in research for decades^35^, its potential to provide policy-relevant insights has been severely constrained by two factors. First, conducting experiments used to require expensive and invasive tools. Second, such studies had to be carried out in a laboratory, which allowed researchers to recruit only relatively homogeneous samples.

However, advances in computer vision now enable researchers to use scalable eye tracking relying on participants’ webcams (“webcam-based eye tracking”)^36^. On the one hand, this approach may make participants less mindful of their eyes being tracked, potentially leading to more natural behavior, thereby increasing the external validity of the results. On the other hand, webcam-based eye tracking enables researchers to recruit large and more heterogeneous samples, thereby enhancing the representativeness of the findings. A growing body of scholarship provides evidence that webcam-based eye tracking is sufficiently accurate to address a wide range of research questions^36–41^.

In light of these developments, eye tracking can now provide valuable insights into the costs and benefits of information provision. For example, if recipients are not influenced by the information provided by policymakers, it could be because they did not see it. Alternatively, recipients may see the information but either disregard it or fail to understand it. In the former case, policymakers need to reconsider how the information is presented, whereas in the latter, they should reconsider the content of the disclosure or, more radically, the viability of transparency-centered interventions. Thus, eye tracking allows policymakers to diagnose the underlying issue, which in turn facilitates finding an appropriate solution.

Moreover, even when information provision produces the desired effects in terms of understanding and behavior, it still imposes a cognitive tax on the recipients. Eye tracking enables researchers to capture an important dimension of this cognitive tax, namely how much time recipients spend paying (visual) attention to the disclosure. All else equal, if recipients require significant time to understand the information, its provision is less likely to be cost-effective, even if it achieves the desired effects.

For Study III, we recruit a sample of 500 U.S. residents on Prolific.co. All participants are paid $1.2 for taking part in the experiment, equivalent to $8.04 per hour. The full protocol of Study III is included in the supplementary information. This study involves remote eye tracking carried out through a widely used platform called i-Motions.

After consenting to participate in the experiment, the participants undergo calibration to ensure proper eye tracking. They are then informed that they will view three fictional smartwatches not currently on the market and will be asked to indicate which one they prefer.

To increase the external validity, we present the information regarding the smart watches in a way that mimics how Apple disclosed information regarding its carbon neutral Apple Watch Series 9. For all three smart watches, we report information about their characteristics, their performance, and their price. For one of the fictitious smart watches, we also provide information about the climate target (carbon neutral).^1^ The order in which respondents see the three smart watches is randomized.

However, one important disclaimer is that the choice made by respondents in this study is not consequential, and financial incentives likely influence attention deployment. Notably, however, we are not interested in absolute levels of attention, as it is well established that salient items in color are likely to attract more attention^42^. Instead, we are interested in the relationship between attention and understanding, specifically, whether treatments that capture more attention lead to a better understanding.

As in Study II, the participants randomly assigned to the control condition only see that the watch is carbon neutral. Participants assigned to the bullet points condition see key characteristics of the carbon neutral target in bullet points format. Participants assigned to the traffic lights condition see information presented using a color-coded traffic lights system.

We defined one AOI for the eye tracking analysis (Fig. 5), ensuring that it is sufficiently large to obtain reliable results, even if our eye tracking technology is less accurate than that used in laboratory studies. We applied inclusive thresholds within the range permitted by the eye-tracking software, requiring data quality above 50% and accuracy of at most $10.0^{\circ }$, to ensure that the data retained for analysis met the minimal acquisition criteria. These thresholds were designed to exclude unsuitable recordings (e.g., those affected by poor brightness or monitor reflections on glasses), thereby maximizing data retention without compromising reliability. The primary dataset was used for the main analyses. As a robustness check, we replicated the analysis, including only participants whose eye-tracking data met higher thresholds, specifically data quality above 80% and accuracy of at most $3.0^{\circ }$, consistent with the criteria applied for AOI tracing (described in the supplementary materials).

Our main variables of interest are TTFF and dwell time (both in absolute terms and in percentage terms). The TTFF of an AOI is the time from the presentation of a stimulus until the AOI is first entered. It captures the conspicuity of the AOI^34^. The dwell time instead indicates the time spent looking at the AOI, and therefore represents the time that a participant has looked at the information contained in the AOI. This can indicate that the participant is either interested in the content of the AOI or is having difficulty extracting information^34^.

Immediately after seeing the three smart watches, participants are asked which one they like the most. Afterwards, we ask respondents: “Carbon neutrality targets have TWO key features. What do you think they are? (maximum 300 characters) Please do not search for the information online. You will be paid even if you do not provide the correct answer! Answer to the best of your ability”. The correct answer is that carbon neutrality does not require emission reduction and only refers to $CO_2$ emissions. We again asked two coders to code the answers to this question in line with Study II. We then ask respondents about their cognitive load, a question on the state of their eyes, and one on whether they are using vision correction. We conclude by asking demographic questions.

## Supplementary Information

Supplementary Information.

## Data Availability

The datasets generated during and/or analysed during the current study are available from the corresponding author on reasonable request.

## Footnotes

1 The smart watch pictures are created with the AI online image generator ’OpenArt’.

## References

[1] Carter, E. Most u.s. consumers don’t know what ’carbon neutral’ means (2022).

[2] ASA. Environmental claims in advertising - qualitative research report. (October 2022).

[3] BEUC. A climate-neutral food basket: Too good to be true. European Consumer Organisation (2023).

[4] Rondoni, A. & Grasso, S. Consumers behaviour towards carbon footprint labels on food: A review of the literature and discussion of industry implications. *Journal of Cleaner Production* **301**, 127031 (2021).

[5] Birkenberg, A., Narjes, M. E., Weinmann, B. & Birner, R. The potential of carbon neutral labeling to engage coffee consumers in climate change mitigation. *Journal of Cleaner Production* **278**, 123621 (2021).

[6] Potter, C. et al. The effects of environmental sustainability labels on selection, purchase, and consumption of food and drink products: A systematic review. *Environment and behavior* **53**, 891–925 (2021). 34456340 10.1177/0013916521995473 PMC8384304

[7] Kim, B.-J., Kim, J.-H. & Yoo, S.-H. Carbon-neutral natural gas in south korea: Households’ perspective obtained through a contingent valuation experiment. *Sustainable Production and Consumption* **33**, 597–607 (2022).

[8] Hartzmark, S. M. & Sussman, A. B. Do investors value sustainability? a natural experiment examining ranking and fund flows. *The Journal of Finance* **74**, 2789–2837 (2019).

[9] Heeb, F., Kölbel, J. F., Paetzold, F. & Zeisberger, S. Do investors care about impact?. *The Review of Financial Studies* **36**, 1737–1787 (2023).

[10] Choisdealbha, Á. N., Timmons, S. & Lunn, P. D. Experimental evidence for the effects of emissions charges and efficiency information on consumer car choices. *Journal of cleaner production* **254**, 120140 (2020).

[11] Panzone, L. A., Sniehotta, F. F., Comber, R. & Lemke, F. The effect of traffic-light labels and time pressure on estimating kilocalories and carbon footprint of food. *Appetite* **155**, 104794 (2020). 32781081 10.1016/j.appet.2020.104794

[12] Dudschig, C., Mackenzie, I. G., Maienborn, C., Kaup, B. & Leuthold, H. Negation and the n400: Investigating temporal aspects of negation integration using semantic and world-knowledge violations. *Language, Cognition and Neuroscience* **34**, 309–319 (2019).

[13] Dudschig, C. & Kaup, B. Can we prepare to negate? negation as a reversal operator. *Journal of Cognition* **3**, 32 (2020). 33043242 10.5334/joc.119 PMC7528695

[14] Dudschig, C. & Kaup, B. How does “not left” become “right”? electrophysiological evidence for a dynamic conflict-bound negation processing account. *Journal of Experimental Psychology: Human Perception and Performance* **44**, 716 (2018). 10.1037/xhp0000481 29154622

[15] Sugden, R. *Valuing environmental preferences. Theory and Practice of the Contingent Valuation Method in the US, EU and Developing Countries* 131–51 (1999) (**Public goods and contingent valuation**).

[16] Modeling the consumer side. van’t Veld, K. Eco-labels. *Annual Review of Resource Economics* **12**, 187–207 (2020).

[17] Enriques, L., Romano, A. & Tuch, A. F. Green gatekeepers. *Minnesota Law Review* **109**, 1 (2025).

[18] Ritov, I. & Kahneman, D. How people value the environment: Attitudes versus economic values. Environment, ethics, and behavior: The psychology of environmental valuation and degradation 33–51 (1997).

[19] Kahneman, D. Daniel kahneman’s gripe with behavioral economics. The Daily Beast 26, (2013) (interview by jesse singal).

[20] Halpern, D. Inside the nudge unit: How small changes can make a big difference (Random House, 2016).

[21] DellaVigna, S. & Linos, E. Rcts to scale: Comprehensive evidence from two nudge units. *Econometrica* **90**, 81–116 (2022).

[22] Szaszi, B. et al. No reason to expect large and consistent effects of nudge interventions. *Proceedings of the National Academy of Sciences* **119**, e2200732119 (2022). 10.1073/pnas.2200732119 PMC9351519 35858388

[23] Gal, D. & Rucker, D. D. Experimental validation bias limits the scope and ambition of applied behavioural science. *Nature Reviews Psychology* **1**, 5–6 (2022).

[24] Dimant, E. & Shalvi, S. Meta-nudging honesty: Past, present, and future of the research frontier. *Current Opinion in Psychology* **47**, 101426 (2022). 35973353 10.1016/j.copsyc.2022.101426

[25] Morvinski, C., Saccardo, S. & Amir, O. Mis-nudging morality. *Management Science* **69**, 464–474 (2023).

[26] Becker, G. M., DeGroot, M. H. & Marschak, J. Measuring utility by a single-response sequential method. *Behavioral science* **9**, 226–232 (1964). 5888778 10.1002/bs.3830090304

[27] Kahneman, D., Knetsch, J. L. & Thaler, R. H. Experimental tests of the endowment effect and the coase theorem. *Journal of political Economy* **98**, 1325–1348 (1990).

[28] Berry, J., Fischer, G. & Guiteras, R. Eliciting and utilizing willingness to pay: Evidence from field trials in northern ghana. *Journal of Political Economy* **128**, 1436–1473 (2020).

[29] West, T. A., Börner, J., Sills, E. O. & Kontoleon, A. Overstated carbon emission reductions from voluntary redd+ projects in the brazilian amazon. *Proceedings of the National Academy of Sciences* **117**, 24188–24194 (2020). 10.1073/pnas.2004334117 PMC7533833 32929021

[30] Battocletti, V., Enriques, L. & Romano, A. The voluntary carbon market: Market failures and policy implications. *U. Colo. L. Rev.* **95**, 519 (2024).

[31] Atal, R. & Sylvan, D. Integrity, equivalence, and imperfect carbon offsets: Implications for carbon markets and net-zero claims. *Institute for Policy Integrity* **1**, 1 (2025).

[32] Nunes, P. A. & Schokkaert, E. Identifying the warm glow effect in contingent valuation. *Journal of Environmental Economics and Management* **45**, 231–245 (2003).

[33] Paas, F. G. & Van Merriënboer, J. J. Variability of worked examples and transfer of geometrical problem-solving skills: A cognitive-load approach. *Journal of educational psychology* **86**, 122 (1994).

[34] Holmqvist, K. Eye tracking: A comprehensive guide to methods and measures (oup, 2011).

[35] Rayner, K. Eye movements in reading and information processing: 20 years of research. *Psychological bulletin* **124**, 372 (1998). 9849112 10.1037/0033-2909.124.3.372

[36] Hutt, S. et al. Webcam-based eye tracking to detect mind wandering and comprehension errors. *Behavior Research Methods* **56**, 1–17 (2024). 36627435 10.3758/s13428-022-02040-x

[37] Xu, P. et al. Turkergaze: Crowdsourcing saliency with webcam based eye tracking. arXiv preprint arXiv:1504.06755 (2015).

[38] Papoutsaki, A., Laskey, J. & Huang, J. Searchgazer: Webcam eye tracking for remote studies of web search. In Proceedings of the 2017 conference on conference human information interaction and retrieval, 17–26 (2017).

[39] Semmelmann, K. & Weigelt, S. Online webcam-based eye tracking in cognitive science: A first look. *Behavior Research Methods* **50**, 451–465 (2018). 28593605 10.3758/s13428-017-0913-7

[40] Valliappan, N. et al. Accelerating eye movement research via accurate and affordable smartphone eye tracking. *Nature communications* **11**, 4553 (2020). 32917902 10.1038/s41467-020-18360-5 PMC7486382

[41] Yang, X. & Krajbich, I. Webcam-based online eye-tracking for behavioral research. *Judgment and Decision making* **16**, 1485–1505 (2021).

[42] Wolfe, J. M. & Horowitz, T. S. Five factors that guide attention in visual search. *Nature human behaviour* **1**, 0058 (2017). 36711068 10.1038/s41562-017-0058 PMC9879335

