# Supplementary material for: Consumer understanding of climate targets is low and hard to improve: evidence from three online studies
Source: Sci Rep. 2026 Sep 11;16:28469. doi: 10.1038/s41598-026-52744-9 (PMC13569486; doi:10.1038/s41598-026-52744-9)
Supplement: Supplementary file 1 [file 41598_2026_52744_MOESM1_ESM.pdf]

1 Sample Composition

1.1 Study I

The sample for Study I consisted of N= 305 participants. The mean age of respondents was 45.93 years (SD = 16.05). The sample was fairly balanced in terms of gender, with 48.4% participants identifying as female (SD = 0.50). Regarding education, the average educational attainment was between a Bachelor's degree and a Master's degree (M = 5.31, SD = 1.92, on a scale where 5 represents a Bachelor's degree and 6 represents a Master's degree). The mean household income category was 5.42 (SD = 3.35), corresponding to an income between $50,000 and $74,999 per year. In terms of political affiliation, 50.3% of participants identified as Democrats (SD = 0.50) and 19.8% as Republicans (SD = 0.40). On a 7-point political ideology scale where higher scores indicated more conservative views, the mean score was 2.86 (SD = 2.01), suggesting a slightly left-of-center average political orientation. Regarding employment status, 64.6% of participants reported being employed (SD = 0.48), while 3.2% identified as students (SD = 0.18). The majority of the sample (73.7%) identified as White (SD = 0.44).

	Mean and Standard Deviation
N	305
Female	0.489 (0.501)
Age	45.93 (16.053)
Income	5.420 (3.345)
Education	5.311 (1.924)
Democrat	0.508 (0.501)
Republican	0.200 (0.401)
Political Scale	2.856 (2.014)
Employed	0.652 (0.477)
Student	0.033 (0.178)
White	0.744 (0.437)

Table 1. The table reports the demographic composition of our sample in Study I.

1.2 Study II

The sample for Study II consisted of N=1,487 participants distributed across four groups: bullet-point (N = 375), Control (N = 370), Information (N = 365), and traffic-lights (N = 377). The gender distribution was relatively balanced across groups and overall, with 49.1% participants identifying as female overall (ranging from 47.7% to 52.9% across groups). The age distribution was also fairly consistent across groups, with the majority of participants (54.1%) falling between 36 and 65 years old. Income levels were diverse, with the most common bracket being $100,000 to $149,999 per household (15.2% overall). In terms of educational attainment 39.3% of participants hold a bachelor's degree and 13.5% hold a master's degree. Politically, 53.4% of respondents identified themselves as Democrats compared to 24.7% Republicans, showing slight variations across groups (50.9% to 55.4% for Democrats). Full-time or part-time employment was reported by 69.3% of participants, with minor variations between groups (67.2% to 71.9%). Only 2.4% of participants identified as students. The majority of participants (77.7%) identified as White, with some variation across groups (73.5% to 81.1%).

	Bullet Points			Control			Group Information			Traffic Lights			Total		
	No.	%	%	No.	%	%	No.	%	%	No.	%	%	No.	%	%
**Gender**															
Other	13	3.5	3.5	12	3.2	3.2	10	2.7	2.7	16	4.2	4.2	51	3.4	3.4
Female	179	47.7	51.2	177	47.8	51.1	193	52.9	55.6	181	48.0	52.3	730	49.1	52.5
Male	183	48.8	100.0	181	48.9	100.0	162	44.4	100.0	180	47.7	100.0	706	47.5	100.0
**Total**	375	100.0		370	100.0		365	100.0		377	100.0		1,487	100.0	
**Age**															
18-25 years old	49	13.1	13.1	44	11.9	11.9	51	14.0	14.0	38	10.1	10.1	182	12.2	12.2
26-35 years old	75	20.0	33.1	79	21.4	33.2	70	19.2	33.2	70	18.6	28.6	294	19.8	32.0
36-45 years old	62	16.5	49.6	47	12.7	45.9	71	19.5	52.6	71	18.8	47.5	251	16.9	48.9
46-55 years old	66	17.6	67.2	72	19.5	65.4	62	17.0	69.6	54	14.3	61.8	254	17.1	66.0
56-65 years old	76	20.3	87.5	78	21.1	86.5	66	18.1	87.7	94	24.9	86.7	314	21.1	87.1
66-75 years old	44	11.7	99.2	43	11.6	98.1	41	11.2	98.9	43	11.4	98.1	171	11.5	98.6
>75 years old	3	0.8	100.0	7	1.9	100.0	4	1.1	100.0	7	1.9	100.0	21	1.4	100.0
**Total**	375	100.0		370	100.0		365	100.0		377	100.0		1,487	100.0	
**Income**															
$10,000 to $19,999	25	6.7	6.7	15	4.1	4.1	24	6.6	6.6	26	6.9	6.9	90	6.1	6.1
$100,000 to $149,999	54	14.4	21.1	58	15.7	19.7	64	17.5	24.1	50	13.3	20.2	226	15.2	21.3
$150,000 or more	44	11.7	32.8	30	8.1	27.8	36	9.9	34.0	28	7.4	27.6	138	9.3	30.5
$20,000 to $29,999	28	7.5	40.3	34	9.2	37.0	26	7.1	41.1	37	9.8	37.4	125	8.4	38.9
$30,000 to $39,999	38	10.1	50.4	32	8.6	45.7	41	11.2	52.3	38	10.1	47.5	149	10.0	49.0
$40,000 to $49,999	39	10.4	60.8	48	13.0	58.6	32	8.8	61.1	37	9.8	57.3	156	10.5	59.4
$50,000 to $59,999	41	10.9	71.7	31	8.4	67.0	33	9.0	70.1	35	9.3	66.6	140	9.4	68.9
$60,000 to $69,999	18	4.8	76.5	31	8.4	75.4	26	7.1	77.3	32	8.5	75.1	107	7.2	76.1
$70,000 to $79,999	36	9.6	86.1	34	9.2	84.6	31	8.5	85.8	36	9.5	84.6	137	9.2	85.3
$80,000 to $89,999	18	4.8	90.9	17	4.6	89.2	21	5.8	91.5	25	6.6	91.2	81	5.4	90.7
$90,000 to $99,999	20	5.3	96.3	23	6.2	95.4	16	4.4	95.9	23	6.1	97.3	82	5.5	96.2
Less than $10,000	14	3.7	100.0	17	4.6	100.0	15	4.1	100.0	10	2.7	100.0	56	3.8	100.0
**Total**	375	100.0		370	100.0		365	100.0		377	100.0		1,487	100.0	
**Education**															
Associate degree in college (2-year)	45	12.0	12.0	39	10.5	10.5	35	9.6	9.6	47	12.5	12.5	166	11.2	11.2
Bachelor's degree in college (4-year)	152	40.5	52.5	150	40.5	51.1	141	38.6	48.2	141	37.4	49.9	584	39.3	50.4
Doctoral degree	5	1.3	53.9	8	2.2	53.2	4	1.1	49.3	14	3.7	53.6	31	2.1	52.5
High school graduate (high school diploma or equivalent including GED)	42	11.2	65.1	53	14.3	67.6	56	15.3	64.7	52	13.8	67.4	203	13.7	66.2
Less than high school degree	5	1.3	66.4	3	0.8	68.4	2	0.5	65.2	2	0.5	67.9	12	0.8	67.0
Master's degree	52	13.9	80.3	46	12.4	80.8	59	16.2	81.4	44	11.7	79.6	201	13.5	80.5
Professional degree (JD, MD)	8	2.1	82.4	8	2.2	83.0	1	0.3	81.6	10	2.7	82.2	27	1.8	82.3
Some college but no degree	66	17.6	100.0	63	17.0	100.0	67	18.4	100.0	67	17.8	100.0	263	17.7	100.0
**Total**	375	100.0		370	100.0		365	100.0		377	100.0		1,487	100.0	
**Political Orientation**															
Republican	96	25.6	25.6	96	25.9	25.9	92	25.2	25.2	83	22.0	22.0	367	24.7	24.7
Democrat	191	50.9	76.5	196	53.0	78.9	198	54.2	79.5	209	55.4	77.5	794	53.4	78.1
Other	88	23.5	100.0	78	21.1	100.0	75	20.5	100.0	85	22.5	100.0	326	21.9	100.0
**Total**	375	100.0		370	100.0		365	100.0		377	100.0		1,487	100.0	
**Employed full time**															
No	123	32.8	32.8	104	28.1	28.1	114	31.2	31.2	116	30.8	30.8	457	30.7	30.7
Yes	252	67.2	100.0	266	71.9	100.0	251	68.8	100.0	261	69.2	100.0	1,030	69.3	100.0
**Total**	375	100.0		370	100.0		365	100.0		377	100.0		1,487	100.0	
**Student**															
No	363	96.8	96.8	362	97.8	97.8	357	97.8	97.8	369	97.9	97.9	1,451	97.6	97.6
Yes	12	3.2	100.0	8	2.2	100.0	8	2.2	100.0	8	2.1	100.0	36	2.4	100.0
**Total**	375	100.0		370	100.0		365	100.0		377	100.0		1,487	100.0	
**White**															
No	73	19.5	19.5	70	18.9	18.9	88	24.1	24.1	100	26.5	26.5	331	22.3	22.3
Yes	302	80.5	100.0	300	81.1	100.0	277	75.9	100.0	277	73.5	100.0	1,156	77.7	100.0
**Total**	375	100.0		370	100.0		365	100.0		377	100.0		1,487	100.0	

Table 2. Balance table: the table reports the demographic composition of our sample in Study II by group and overall.

1.3 Study III

The sample for Study III consisted of N=475 participants, divided into three treatment groups: Control (N = 168, 35.4%), bullet-point (N = 147, 30.9%), and traffic-lights (N = 160, 33.7%). Gender distribution varied slightly across groups, with females comprising 51.2% of the Control group, 40.8% of the bullet-point group, and 51.9% of the traffic-lights group. The majority (66.7%) of participants across all groups had an age range between 26 and 55 years old. Income levels were also balanced across groups, with the most common bracket per household being $100,000 to $149,999 (19.2% overall). Educational attainment was high across all groups, with Bachelor's degrees being most common (39.1% overall), though the bullet-point group had a slightly higher proportion of participants with graduate degrees (27.4% vs. 16.7% in Control and 17.6% in the traffic-lights group). Politically, all groups leaned Democratic (51.6% overall), with the Control and Traffic groups showing slightly higher proportions of Democrats (52.4% and 52.5% respectively) compared to the bullet-point group (49.7%). The bullet-point group had the highest proportion of Republicans (21.8% vs. 15.5% in Control and 18.8% in the traffic-lights group). Employment rates were similar in the Control and bullet-point groups (72.6% and 73.5% respectively) but lower in the traffic-lights group (66.9%). Student representation was higher in the bullet-point and Traffic groups (6.8% and 6.2%) compared to the Control group (3.0%). Racial composition showed some variation, with white participants comprising 70.2% of the Control group, 60.5% of the bullet-point group, and 67.5% of the traffic-lights group. These demographic differences across treatment groups, while generally minor, should be considered when interpreting the results of the study.

	Stimulus											
	Control			Bullet Points			Traffic Lights			Total		
	No.	%	%	No.	%	%	No.	%	%	No.	%	%
**Gender**												
Other	7	4.2	4.2	6	4.1	4.1	4	2.5	2.5	17	3.6	3.6
Female	86	51.2	55.4	60	40.8	44.9	83	51.9	54.4	229	48.2	51.8
Male	75	44.6	100.0	81	55.1	100.0	73	45.6	100.0	229	48.2	100.0
**Total**	168	100.0		147	100.0		160	100.0		475	100.0	
**Age**												
18-25 years old	15	8.9	8.9	18	12.2	12.2	20	12.5	12.5	53	11.2	11.2
26-35 years old	49	29.2	38.1	46	31.3	43.5	47	29.4	41.9	142	29.9	41.1
36-45 years old	50	29.8	67.9	33	22.4	66.0	39	24.4	66.2	122	25.7	66.7
46-55 years old	25	14.9	82.7	20	13.6	79.6	27	16.9	83.1	72	15.2	81.9
56-65 years old	22	13.1	95.8	21	14.3	93.9	12	7.5	90.6	55	11.6	93.5
66-75 years old	7	4.2	100.0	7	4.8	98.6	14	8.8	99.4	28	5.9	99.4
>75 years old	0	0.0	100.0	2	1.4	100.0	1	0.6	100.0	3	0.6	100.0
**Total**	168	100.0		147	100.0		160	100.0		475	100.0	
**Q12**												
$10,000 to $19,999	5	3.0	3.0	5	3.4	3.4	11	6.9	6.9	21	4.4	4.4
$100,000 to $149,999	34	20.2	23.2	29	19.9	23.3	28	17.6	24.5	91	19.2	23.7
$150,000 or more	20	11.9	35.1	23	15.8	39.0	19	11.9	36.5	62	13.1	36.8
$20,000 to $29,999	15	8.9	44.0	13	8.9	47.9	10	6.3	42.8	38	8.0	44.8
$30,000 to $39,999	10	6.0	50.0	11	7.5	55.5	14	8.8	51.6	35	7.4	52.2
$40,000 to $49,999	15	8.9	58.9	7	4.8	60.3	19	11.9	63.5	41	8.7	60.9
$50,000 to $59,999	21	12.5	71.4	14	9.6	69.9	16	10.1	73.6	51	10.8	71.7
$60,000 to $69,999	14	8.3	79.8	12	8.2	78.1	12	7.5	81.1	38	8.0	79.7
$70,000 to $79,999	12	7.1	86.9	13	8.9	87.0	7	4.4	85.5	32	6.8	86.5
$80,000 to $89,999	8	4.8	91.7	10	6.8	93.8	3	1.9	87.4	21	4.4	90.9
$90,000 to $99,999	4	2.4	94.0	5	3.4	97.3	12	7.5	95.0	21	4.4	95.3
Less than $10,000	10	6.0	100.0	4	2.7	100.0	8	5.0	100.0	22	4.7	100.0
**Total**	168	100.0		146	100.0		159	100.0		473	100.0	
**Q15**												
Associate degree in college (2-year)	21	12.5	12.5	13	8.9	8.9	17	10.7	10.7	51	10.8	10.8
Bachelor's degree in college (4-year)	69	41.1	53.6	50	34.2	43.2	66	41.5	52.2	185	39.1	49.9
Doctoral degree	3	1.8	55.4	8	5.5	48.6	3	1.9	54.1	14	3.0	52.9
High school graduate (high school diploma or equivalent including GED)	20	11.9	67.3	12	8.2	56.8	18	11.3	65.4	50	10.6	63.4
Less than high school degree	1	0.6	67.9	1	0.7	57.5	0	0.0	65.4	2	0.4	63.8
Master's degree	22	13.1	81.0	25	17.1	74.7	24	15.1	80.5	71	15.0	78.9
Professional degree (JD, MD)	3	1.8	82.7	7	4.8	79.5	1	0.6	81.1	11	2.3	81.2
Some college but no degree	29	17.3	100.0	30	20.5	100.0	30	18.9	100.0	89	18.8	100.0
**Total**	168	100.0		146	100.0		159	100.0		473	100.0	
**Political Orientation**												
Republican	26	15.5	15.5	32	21.8	21.8	30	18.8	18.8	88	18.5	18.5
Democrat	88	52.4	67.9	73	49.7	71.4	84	52.5	71.2	245	51.6	70.1
Other	54	32.1	100.0	42	28.6	100.0	46	28.7	100.0	142	29.9	100.0
**Total**	168	100.0		147	100.0		160	100.0		475	100.0	
**employed**												
No	46	27.4	27.4	39	26.5	26.5	53	33.1	33.1	138	29.1	29.1
Yes	122	72.6	100.0	108	73.5	100.0	107	66.9	100.0	337	70.9	100.0
**Total**	168	100.0		147	100.0		160	100.0		475	100.0	
**student**												
No	163	97.0	97.0	137	93.2	93.2	150	93.8	93.8	450	94.7	94.7
Yes	5	3.0	100.0	10	6.8	100.0	10	6.2	100.0	25	5.3	100.0
**Total**	168	100.0		147	100.0		160	100.0		475	100.0	
**white**												
No	50	29.8	29.8	58	39.5	39.5	52	32.5	32.5	160	33.7	33.7
Yes	118	70.2	100.0	89	60.5	100.0	108	67.5	100.0	315	66.3	100.0
**Total**	168	100.0		147	100.0		160	100.0		475	100.0	

Table 3. Balance table: the table reports the demographic composition of our sample in Study III by group and overall.

2 Summary statistics

2.1 Study I

.

Correct Differences	
Observations	305
Mean	0.1016
Median	0
Standard Deviation	0.3027
Minimum	0
Maximum	1

Table 4. Summary Statics (Mean, Median, Standard Deviation, Minimum and Maximum) for the variable capturing whether participants identified the two correct differences between carbon neutrality and net-zero claims in Study I.

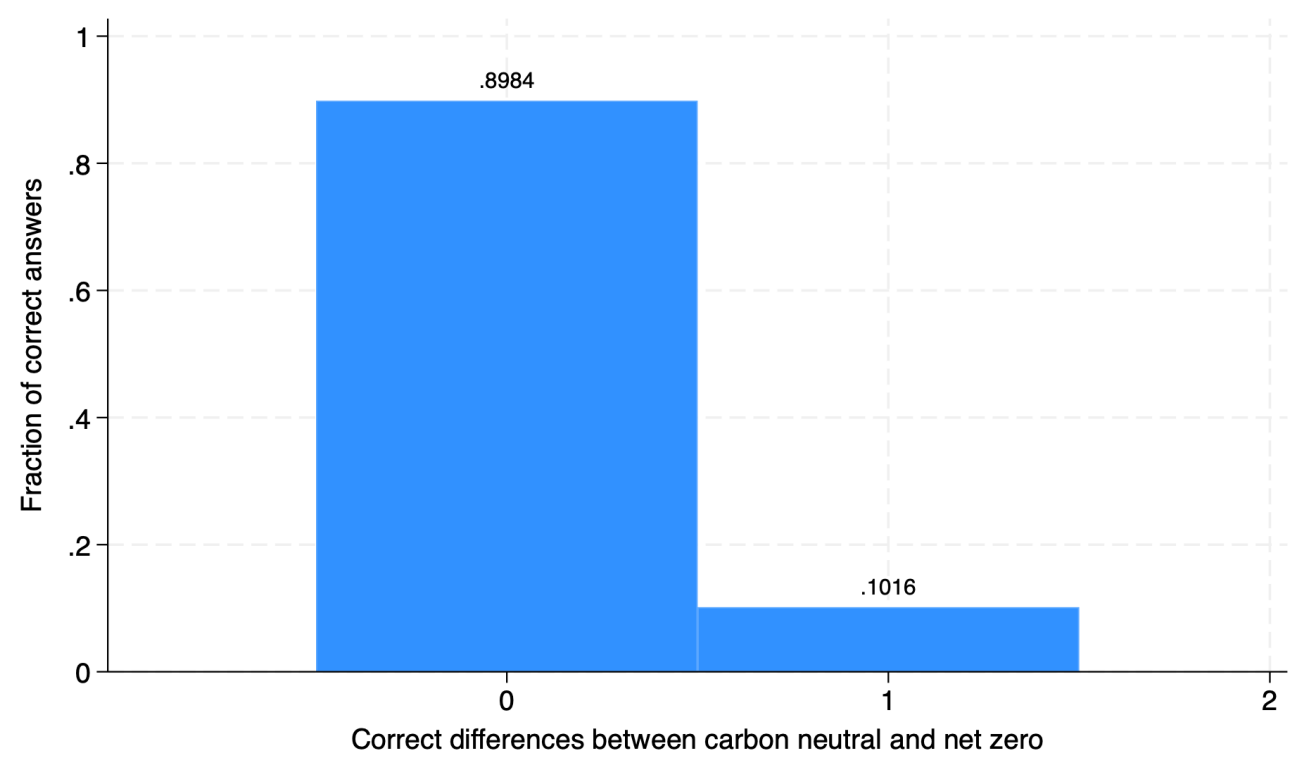

Figure 1. The fraction of participants who correctly identified the differences between net-zero and carbon neutrality in Study I. The numbers above the bar show the proportion of participants who received a score of 0, 1 or 2 in this question.

2.2 Study II

.

	(1) All		(2) Control		(3) Information		(4) Bullet Points		(5) Traffic Lights	
	mean	sd	mean	sd	mean	sd	mean	sd	mean	sd
Visual Recognition	3.95	1.98	3.02	1.46	3.73	1.84	4.78	2.06	4.24	2.05
Unprompted understanding	0.45	0.55	0.32	0.47	0.51	0.55	0.51	0.58	0.46	0.55
Total Understanding	4.40	2.16	3.34	1.53	4.24	1.98	5.29	2.29	4.70	2.27
Observations	1487		370		365		375		377	

Table 5. Mean and standard deviation for the questions used to assess the participants' understanding by group in Study II.

	Group									
	**All**		**Control**		**Information**		**Bullet Points**		**Traffic Lights**	
	mean	sd	mean	sd	mean	sd	mean	sd	mean	sd
WTP carbon neutral	6.49	6.11	7.03	6.19	6.30	6.12	6.19	5.72	6.46	6.41
WTP net zero	7.15	6.35	7.18	6.31	7.09	6.07	6.57	5.87	7.75	7.05
WTP Difference	0.65	3.99	0.15	3.87	0.79	3.41	0.38	4.37	1.30	4.15
Observations	1487		370		365		375		377	

Table 6. Mean and standard deviation for the questions used to assess the participants' WTP by group in Study II.

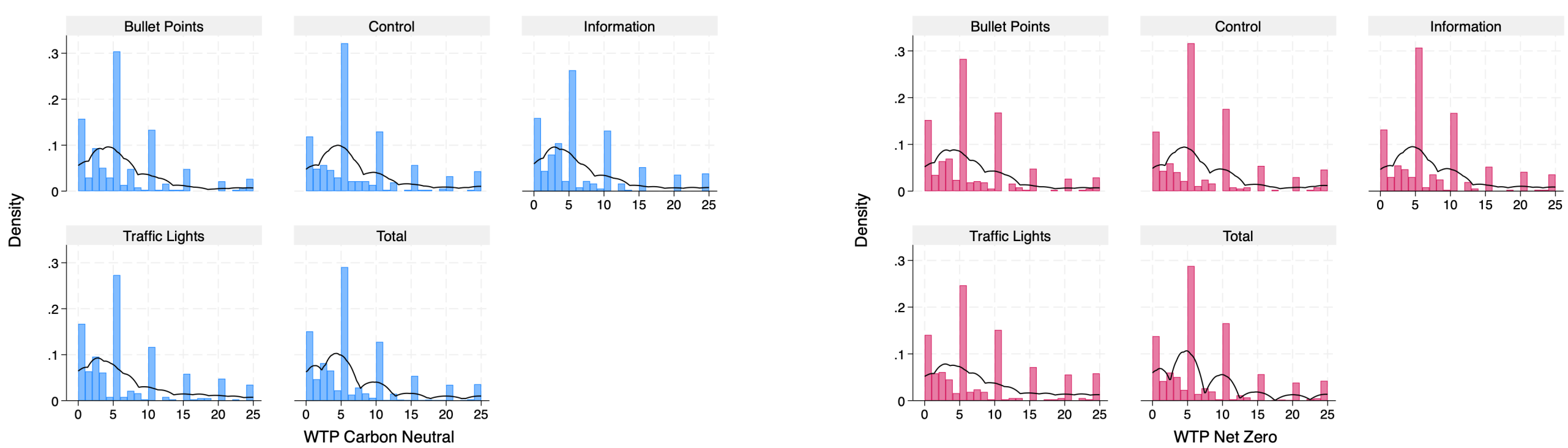

Figure 2. Density distribution of the WTP for carbon neutral (left) and net-zero (right) across the treatment groups and in total.

	(1)		(2)		(3)		(4)		(5)	
	All		Control		Info		Bullet Points		Traffic Lights	
	mean	sd	mean	sd	mean	sd	mean	sd	mean	sd
Consistency	0.57	0.50	0.62	0.49	0.58	0.49	0.50	0.50	0.58	0.49
Warm Glow Carbon Neutral	2.43	1.09	2.53	1.07	2.37	1.05	2.40	1.12	2.40	1.10
Warm Glow Net-Zero	2.50	1.10	2.48	1.10	2.52	1.07	2.45	1.11	2.56	1.13
Cognitive load	5.10	1.59	4.99	1.67	5.37	1.56	4.94	1.54	5.11	1.57
Info Avoidance: Life	1.84	0.75	1.85	0.75	1.83	0.74	1.86	0.74	1.80	0.77
Info Avoidance: Alzheimer	2.20	0.76	2.24	0.76	2.17	0.76	2.21	0.77	2.17	0.76
Info Avoidance: Stress	2.16	0.74	2.13	0.75	2.15	0.73	2.18	0.73	2.17	0.76
Worry about Climate Change (PCA)	-0.00	1.57	0.06	1.50	0.01	1.57	-0.10	1.62	0.04	1.57
Info Avoidance (PCA)	0.00	1.43	0.06	1.45	0.00	1.41	-0.01	1.42	-0.06	1.44
Observations	1487		370		365		375		377	

Table 7. Mean and standard deviation for the questions used to assess the mechanisms driving the results by group in Study II.

2.3 Study III

.

	(1)		(2)		(3)		(4)	
	All		Control		Bullet Points		Traffic Lights	
	mean	sd	mean	sd	mean	sd	mean	sd
Wrong Answers	0.21	0.41	0.18	0.39	0.18	0.38	0.29	0.45
Observations	500		168		146		159	

Table 8. Summary statistics (Mean, Standard Deviation) for the wrong answers given to the open-ended question overall and in each condition.

**Measure**	**Control**	**Bullet points**	**Traffic lights**
Mean	0.185 (0.030)	0.178 (0.032)	0.289 (0.036)
Difference from Control	-	-0.006 (0.044)	-0.104** (0.047)

Table 9. Summary statistics (mean and standard errors in parentheses) for answers that contradict the definition of carbon neutrality across the treatment groups in Study III. The p-values reported are all for a two-sided paired t-tests with unequal variances. Standard errors are reported in parentheses next to the mean and approximated to the third digit.

	(1)		(2)		(3)		(4)	
	All		Control		Bullet Points		Traffic Lights	
	mean	sd	mean	sd	mean	sd	mean	sd
Revisit times	0.63	1.09	0.59	1.02	0.61	0.98	0.68	1.21
Fixation count	2.41	2.22	2.21	2.29	2.63	2.33	2.38	2.10
Time to first fixation	7935.18	5605.35	7858.79	4662.13	9661.56	5782.67	6730.50	5807.58
Dwell time	2821.53	3935.59	1320.68	1576.82	2594.91	2754.25	4024.23	5240.78
Dwell time spent on AOI (%)	14.01	14.73	7.28	6.86	13.33	13.65	19.16	17.40
Observations	196		56		59		81	

Table 10. Summary Statistics (Mean, Standard Deviation) for the variables (Revisits, Fixation count, TTFF, Dwell Time, Dwell time spent on the AOI expressed in percentage) capturing participants' attention to the AOI overall and in each condition in Study III.

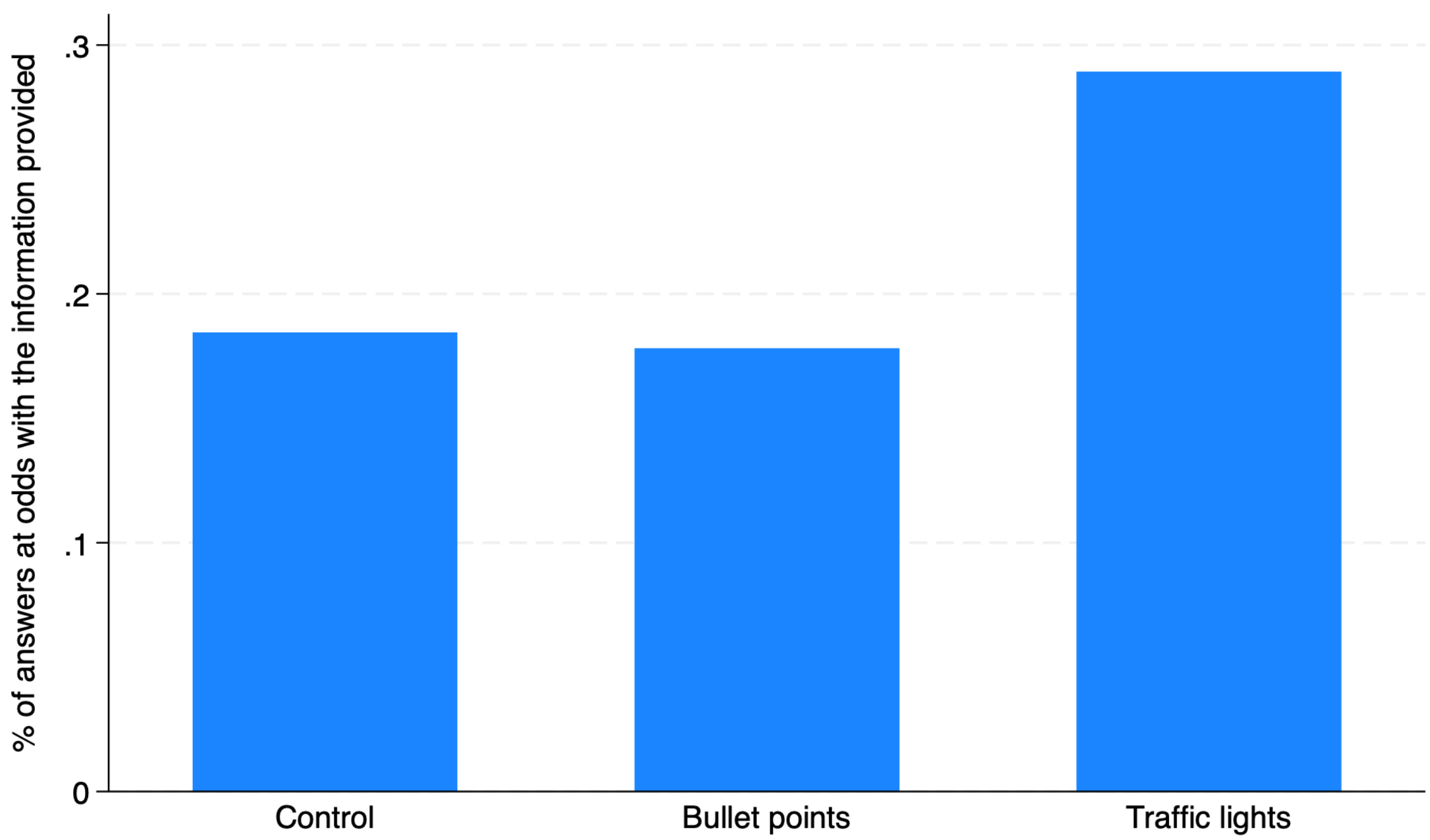

Figure 3. Percentage of participants who incorrectly associate carbon neutrality to either emission reduction, low emission or no emission by group.

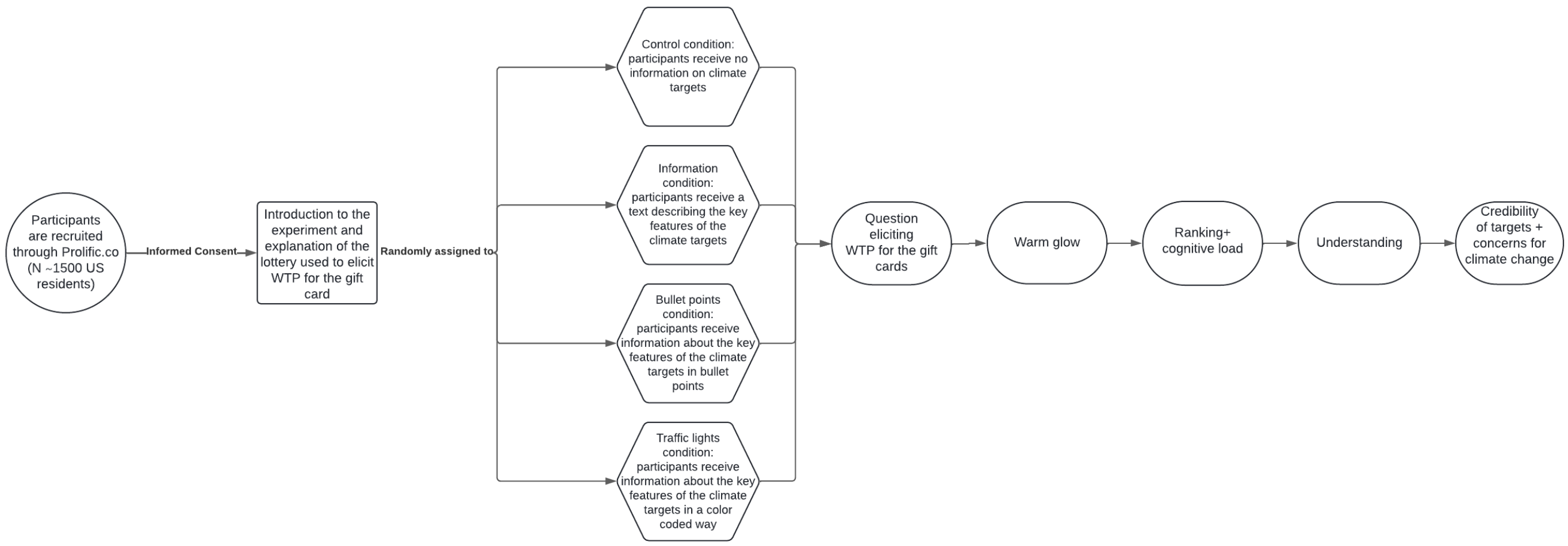

Figure 4. Experimental flow of Study II.

2.4 Study 2 Protocol

.

At the beginning of the experiment, all respondents see the same text explaining that many firms are setting climate targets like carbon neutrality and net-zero, and that we are interested in knowing how much they value these claims. We then explain to respondents that by taking part in the experiment, they automatically entered a lottery with the possibility to win a $25 gift card from a company with no climate target and $25 cash via Prolific.

Participants are informed that they would be asked two questions to state how much of the $25 cash they might win they would be willing to pay in order to switch from the gift card from a company without a climate target to: *i*) the gift card from a company with a net-zero target, *ii*) the gift card from a company with a carbon neutral target. The order of these two questions is randomized.

The precise instructions received by participants are "*We will ask you two questions. In each question we will ask you how much you would be willing to pay from the $25 cash you may win to receive a gift card from a company with a climate target instead of a gift card from a company with no climate target. If you win the lottery, we will select at random one of the questions. Based on the answer you provided to the randomly selected question, you may end up paying the amount you state or a lower one from your cash winnings to receive the gift card from the company with a climate target. A price between $0 and $25 will be selected at random for the gift card from the company with a climate target. If you have indicated that you are willing to pay more than the randomly drawn price and you win the lottery, you will receive a $25 gift card from the company with a given target and $25 minus the price drawn for the gift card.*

If you have indicated that you are willing to pay less than the randomly drawn price and win the lottery, you will receive a $25 gift card for a company with no climate target and $25 cash.

Important: The value of the gift card will always be $25, so if you were to win the lottery, you could receive either:

- *A) A $25 gift card from a company with no climate target and $25 cash, or*
- B) *A $25 gift card from a company that has a climate target but less than $25 cash*

Notice the following two things: (1) Your decision has no effect on the randomly selected price and (2) It is in your interest to indicate your true preferences when answering each of the questions."

Question	Range	Level
There are TWO key differences between a carbon neutral company and a net-zero company. What do you think they are? (maximum 300 characters) Remember not to search for information online. You will be paid even if you do not provide the correct answer! Answer to the best of your ability.	Open-ended - 300 characters max	Unprompted understanding
You have seen the gift cards of two companies with a climate target. One company had a carbon neutral target and one had a net-zero target. Can you assign the following features to the right gift card based on what you have seen?	8 features: Emissions' cut required, Committed (not yet achieved), No emissions' cut required, Already achieved, Covers all GHGs emissions, Covers only CO2 emissions, Doesn't use energy from coal, Doesn't use energy from fossil fuels	Visual recognition

Table 11. Understanding questions for Study II.

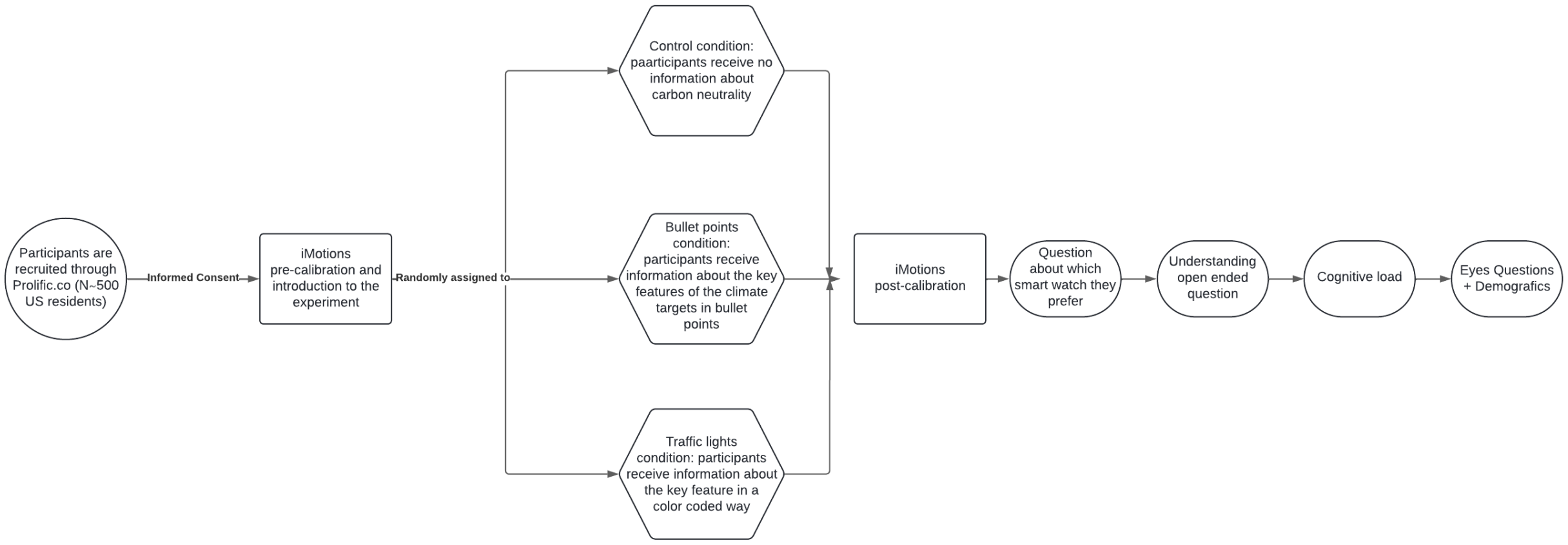

Figure 5. Experimental flow of Study III.

2.5 Study III Flow

	SBTI
Net-Zero Definition	A state of balance between anthropogenic emissions and anthropogenic removals. In most cases, it is important to specify either net-zero CO2 emissions or net-zero GHG emissions, which also includes non-CO2 GHGs. Net-zero GHG emissions must be achieved at the global level to stabilize temperature increase, and targets set using the net-zero Standard must cover all UNFCCC/Kyoto GHG emissions. The SBTi's net-zero Standard outlines what companies need to do to enable the global economy to achieve net-zero. The Standard makes clear that for corporate net-zero targets in line with keeping global warming to 1.5°C require rapid and deep emission reductions. Companies must take action to halve their emissions by around 2030. Likewise, long-term deep emissions cuts of at least 90% before 2050 are crucial for net-zero targets to align with science.
Carbon neutral definition	Defines carbon neutrality with reference to net-zero
Differences	Although often used interchangeably with 'net-zero', the two are not the same. In general, when companies claim carbon neutrality they are counterbalancing CO2 emissions with carbon offsets without necessarily having reduced emissions by an amount consistent with reaching net-zero at the global or sector level. This may conceal the need for deeper emissions reductions that are in line with what the science requires for the world to keep global warming to 1.5°C. Carbon neutrality claims also do not necessarily cover non-CO2 GHGs
C02 vs GHG	Yes, it explicitly states that carbon neutrality does not necessarily cover non-CO2 GHGs
Emission reductions	Yes, it flags that carbon neutrality does not imply emission reductions. Net-zero requires emission reductions.
Overall consistent with the difference flagged in the study	Yes

Table 12. Science Based Targets initiative definitions of net-zero and carbon neutrality.

2.6 Definitions

We reported the definitions of net-zero and carbon neutrality provided by SBTi, the UN and ISO. Similarly to the SBTi, they highlighted that unlike carbon neutrality, net-zero pledges necessarily required deep emission reductions and covered all GHGs emissions.

	ISO
Net-Zero Definition	"a condition in which human-caused residual greenhouse gas (GHG) emissions are balanced by human-led removals over a specified period and within specified boundaries". Achieving this balance is a complex process involving emission reductions, offsets and innovative technologies
Carbon Neutral definition	Businesses often speak about becoming "carbon neutral". It means they're taking steps to remove the equivalent amount of CO2 to what is emitted through activities across their supply chains. This operation is called offsetting and allows organizations to continue doing business with a good conscience.
Differences	Net-zero goes beyond carbon neutrality, encompassing all GHGs, including methane (CH4), nitrous oxide (N2O) and other hydrofluorocarbons, which tend to trap more heat than carbon dioxide. It consists of ambitious emission reductions, systemic changes across sectors and active removal of emissions.
C02 vs GHG	Yes, it explicitly mentions CO2 when discussing carbon neutrality and all GHGs when discussing net-zero
Emission reductions	Yes, it flags ambitious emissions reductions when talking about net-zero. No mention of emission reductions when talking about carbon neutrality.
Overall consistent with the difference flagged in the study	yes

Table 13. ISO definition of net-zero and carbon neutrality

	United Nations
Net-Zero Definition	net-zero means cutting greenhouse gas emissions to as close to zero as possible, with any remaining emissions re-absorbed from the atmosphere, by oceans and forests for instance
Carbon Neutral definition	Carbon neutrality, or having a net-zero carbon footprint, refers to achieving net-zero carbon emissions by balancing a measured amount of carbon released with an equivalent amount sequestered or offset.
Differences	Not explicitly stated
C02 vs GHG	Yes, in the definition of net-zero it mentions all GHGs, whereas in the definition of carbon neutrality only mentions CO2
Emission reductions	Yes, net-zero definition implies cutting greenhouse gas emissions, whereas emission reductions are not mentioned for carbon neutrality
Overall consistent with the difference flagged in the study	yes

Table 14. United Nations definition of net-zero and carbon neutrality

3 Eye Tracking Specifications

We describe the specifications of the eye-tracking methodology used in this study following the guidelines outlined by[1]. This study employed a video-based tracking algorithm to analyses data collected remotely via webcam-based acquisition. The respondents' video and screen recordings were obtained through a browser-based platform (iMotions, Copenhagen, Denmark). Thus, the recording apparatus was dependent on each respondent's setup. The recordings were analyzed using the WebET 3.0 eye-tracking algorithm (iMotions, Copenhagen, Denmark), which provided gaze mapping by extracting respondent data through the iMotions software (version 10). The raw Cartesian coordinates of the gaze points were then processed using a velocity-based event classification system to identify fixations and saccades. Remote data collection precluded direct control over acquisition conditions and respondents' compliance. Therefore, the head movement were unconstrained and lighting conditions could not be standardized. To address the first issue, the acquisition process was preceded by a head position check, as the participants were invited to place their head in the correct position in order to begin the experiment. The head position check aimed at ensuring: i) that all participants maintained a distance from the screen of approximately 65 centimeters, ii) a more uniform visual angle projection across respondents. Moreover, if participants moved too much from the ideal position, the software automatically started an additional head position check. Regarding lighting condition, the platform provided instructions to participants on how to optimize their setup. These instructions specified the arrangement of the participant and camera in relation to light sources and room illumination, advising against backlighting and promoting direct facial illumination. However, we could not verify whether respondents adhered to these guidelines. Furthermore, the webcam-based eye-tracking algorithm for event classification was based on a processing pipeline that included firstly face detection and localization, eye detection within the face, and eye orientation detection, followed by gaze orientation mapping onto the screen through a calibration procedure. Eye-tracking data acquisition followed a multi-step process centered around the calibration phase. The system-controlled calibration procedure, validated by the tool manufacturer, involved the presentation of 13 calibration points at both the beginning and end of the testing session. Calibration determined how gaze orientation was mapped onto the screen and played a fundamental role in linking eye movement patterns to scene exploration, as well as providing key information on tracking accuracy. The calibration accuracy value was one of the criteria for participant inclusion in filtered sample for the analysis, alongside an evaluation of overall data quality. This combination of measures helped to exclude participants whose recordings exhibited reduced precision and a lower number of gaze detections, which could indicate inadequate recording conditions and/or non-compliance with the procedure, leading to unreliable representations of gaze direction. As anticipated in the main body of the manuscript, the refined dataset was defined using predefined thresholds: only data from participants with an eye-tracking accuracy of less than 3.0 visual degrees and an overall dataset quality of at least 80% were included. This approach indirectly accounted for potential uncontrolled interferences during data collection. Moreover, it helped compensate for the reduced sampling frequency inherent to remote webcam-based acquisition, which was found to be approximately 60 milliseconds. Visual attention results were derived through an Areas of Interest (AOIs) analysis of the gaze data. Although AOIs can be fundamental in gaze mapping analysis, there remains a lack of consensus regarding their optimal definition, despite their characteristics directly influencing analytical outcomes[2]. It has therefore been suggested that AOI determination should prioritize objectivity and reproducibility, also to enhance the replicability of findings[3]. AOI delineation methods have been classified by[4] into three categories: shape-based, data-driven, and stimulus-based approaches, with hybrid methods also being applied. The shape-based AOIs refer to predefined regions within the visual scene that are independent of specific items or gaze data. These are better suited for assessing general visual exploration patterns rather than pinpointing visual attention loci. The data-driven AOIs are determined based on participants' gaze behavior, making them highly dependent on the sample under investigation and less representative of the actual scene elements. The stimulus-based AOIs allow for sample-independent tracing, as they are delineated based on the characteristics of the objects under analysis - including native shape, size, spatial position, and saliency attributes. Ideally, an AOI should be designed to capture all fixations (or relevant eye-tracking metrics) associated with a specific object, while excluding those related to adjacent elements[5]. While some have advocated for maximizing AOI size, based on the premise that gaze tends to cluster around informative areas[6], this approach presents challenges when multiple salient objects are present in close proximity within the scene. Consequently, the delineation of AOIs around the objects under analysis should be carefully managed to avoid the exclusion of gaze points that are specifically related to the object itself (AOIs too small) while also preventing the inclusion of gaze points from neighboring objects (AOIs too large and/or overlapping). A key aspect in AOI definition was therefore the consideration of eye-tracking system accuracy, as this has been reported to play a crucial role in enhancing the sensitivity and specificity of AOI-based analysis[7]. Therefore, the AOIs were designed to fully encompass the items under consideration within the presented visual scenes while accounting for the dispersion of gaze points inherent to the eye-tracking system, while specific adaptations were introduced where necessary to prevent any overlap of AOI boundaries that could lead to confounding effects. The AOIs were initially delineated to align with the native shape of the objects and to include the maximum vertical and horizontal projections of their geometric dimensions. The contour of each AOI was then expanded by an amount corresponding to 1.5 degrees of visual angle, applied horizontally and vertically, or radially - depending on the shape of the object. This adjustment was introduced to enhance specificity and

reduce Type II errors (false negatives) by ensuring that relevant gaze points were not inadvertently excluded due to the inherent accuracy limitations of the eye-tracking system. To further improve specificity and minimize Type I errors (false positives), a fixed distance between AOIs was maintained by leaving a minimum spacing corresponding to 1.0 degree of visual angle. This accounted for the range within which the algorithm transduced gaze points into fixations, thereby reducing the likelihood of erroneous fixation assignment to adjacent AOIs. Finally, a visual angle of 3.0 degrees was used as a reference both for defining AOI expansion and as an accuracy threshold for filtering the dataset to include only participants with higher-quality eye-tracking data. This dual-purpose approach enabled a robustness check of the eye-tracking analysis, comparing results between the filtered dataset and the full sample. The consistency of findings across both datasets confirmed the stability of the results and the reliability of AOI determination.

4 Regressions

4.1 Study I

	(1) Correct Differences	(2) Correct Differences	(3) Correct Differences
Correct Differences			
Education		-0.198*	
		(0.070)	
Age		0.00797	0.00467
		(0.437)	(0.694)
Female		-0.414	-0.483
		(0.290)	(0.231)
Income		0.0354	
		(0.531)	
Democrat		0.214	0.0236
		(0.586)	(0.965)
White			-0.404
			(0.342)
Political Scale			-0.0743
			(0.565)
Republican			0.0415
			(0.953)
$10,000 to $19,999			1.597
			(0.339)
$20,000 to $29,999			1.189
			(0.476)
$30,000 to $39,999			0.219
			(0.910)
$40,000 to $49,999			1.326
			(0.417)
$50,000 to $59,999			1.171
			(0.507)
$60,000 to $69,999			2.288
			(0.171)
$70,000 to $79,999			0.786
			(0.660)
$80,000 to $89,999			0.543
			(0.757)
$90,000 to $99,999			1.037
			(0.556)
$100,000 to $149,999			1.670
			(0.294)
$150,000 or more			0.154
			(0.935)
Some college but no degree			-3.791***
			(0.002)
College (2-year)			-3.778***
			(0.002)
College (4-year)			-3.070***
			(0.009)
Master's degree			-2.972**
			(0.012)
Professional degree (JD, MD)			-3.234*
			(0.054)
High school graduate			-5.170***
			(0.001)
Constant	-2.179***	-1.670*	0.559
	(0.000)	(0.053)	(0.694)
Observations	305	305	287
Pseudo R^2	0.000	0.024	0.124
chi2		5.393	26.93

p-values in parentheses

* $p < 0.10$, ** $p < 0.05$, *** $p < 0.01$

Table 15. Determinants of correct differences in Study I. All regressions are run through a logit model with robust standard errors. We note that the results on different qualifications are not robust and measured on a sample where 117 out of 300 participants have a Bachelor's degree in college (4-year).

4.2 Study II

	(1) WTP CN	(2) WTP Net-Zero	(3) Tot Understanding	(4) Multiple Choice	(5) Open Question
Control	1	1	1	1	1
Bullet Points	1.829	1.572	73.42***	58.80***	8.967**
	(0.470)	(0.607)	(0.000)	(0.000)	(0.025)
Information	0.210	0.784	13.14***	11.82***	2.745
	(0.159)	(0.802)	(0.000)	(0.001)	(0.278)
Traffic Lights	0.627	0.209	7.100**	7.342**	1.919
	(0.635)	(0.123)	(0.017)	(0.013)	(0.476)
Warm Glow CN	2.719***	1.222	0.777	0.843	0.674*
	(0.000)	(0.299)	(0.143)	(0.331)	(0.096)
Warm Glow Net-Zero	1.047	2.606***	1.367**	1.333*	1.203
	(0.806)	(0.000)	(0.035)	(0.063)	(0.387)
Bullet Points × Warm Glow CN	0.761	0.758	1.412	1.378	1.365
	(0.316)	(0.299)	(0.220)	(0.246)	(0.331)
Information × Warm Glow CN	1.249	1.043	0.884	0.792	1.532
	(0.406)	(0.870)	(0.616)	(0.347)	(0.167)
Traffic Lights × Warm Glow CN	0.722	0.884	1.499	1.440	1.322
	(0.264)	(0.629)	(0.127)	(0.168)	(0.339)
Bullet Points × Warm Glow Net-Zero	1.291	1.163	0.651	0.612*	1.018
	(0.389)	(0.615)	(0.123)	(0.074)	(0.955)
Information × Warm Glow Net-Zero	0.898	0.878	0.999	0.983	1.053
	(0.711)	(0.656)	(0.997)	(0.947)	(0.863)
Traffic Lights × Warm Glow Net-Zero	1.307	1.186	0.818	0.845	0.859
	(0.358)	(0.514)	(0.482)	(0.545)	(0.580)
Cognitive Load	1.142*	1.047	0.943	0.937	0.981
	(0.064)	(0.551)	(0.330)	(0.289)	(0.810)
Bullet Points × Cognitive Load	0.828*	0.975	0.923	0.926	0.997
	(0.062)	(0.809)	(0.472)	(0.510)	(0.980)
Information × Cognitive Load	0.962	0.937	0.954	0.981	0.933
	(0.748)	(0.530)	(0.589)	(0.833)	(0.567)
Traffic Lights × Cognitive Load	0.918	1.161	1.106	1.123	0.991
	(0.446)	(0.204)	(0.331)	(0.269)	(0.936)
Info Avoidance (PCA)	0.901	0.922	1.027	1.019	1.050
	(0.182)	(0.338)	(0.705)	(0.792)	(0.618)
Bullet Points × Info Avoidance (PCA)	0.992	0.903	1.178	1.125	1.180
	(0.945)	(0.412)	(0.161)	(0.325)	(0.230)
Information × Info Avoidance (PCA)	1.049	0.953	1.038	1.021	1.070
	(0.680)	(0.674)	(0.731)	(0.857)	(0.610)
Traffic Lights × Info Avoidance (PCA)	1.084	1.085	1.012	1.018	0.984
	(0.496)	(0.512)	(0.918)	(0.870)	(0.902)
Total Understanding	0.927	0.980			
	(0.279)	(0.794)			
Bullet Points × Total Understanding	1.062	1.048			
	(0.479)	(0.605)			
Information × Total Understanding	1.026	0.980			
	(0.779)	(0.830)			
Traffic Lights × Total Understanding	1.025	0.989			
	(0.791)	(0.908)			
Bullet Points × Credibility CN	1.044	1.061	0.781	0.886	0.517***
	(0.822)	(0.742)	(0.106)	(0.434)	(0.001)
Control × Credibility CN	1.214	1.170	1.354*	1.360*	1.023
	(0.252)	(0.390)	(0.065)	(0.058)	(0.913)
Information × Credibility CN	2.072***	1.511**	0.673**	0.695**	0.784
	(0.000)	(0.018)	(0.012)	(0.035)	(0.247)
Traffic Lights × Credibility CN	1.031	0.967	0.808	0.814	0.918
	(0.881)	(0.853)	(0.237)	(0.248)	(0.635)
Bullet Points × Credibility Net-Zero	1.033	0.928	0.797	0.813	0.898
	(0.857)	(0.669)	(0.204)	(0.252)	(0.596)
Control × Credibility Net-Zero	0.944	0.991	1.008	0.966	1.264
	(0.708)	(0.957)	(0.952)	(0.798)	(0.176)
Information × Credibility Net-Zero	0.963	1.165	1.207	1.212	0.990
	(0.811)	(0.307)	(0.182)	(0.216)	(0.957)
Traffic Lights × Credibility Net-Zero	1.435	1.596**	0.787	0.720*	1.182
	(0.122)	(0.018)	(0.199)	(0.074)	(0.343)
Consistency	0.695***	1.088	1.285**	1.239*	1.281*
	(0.003)	(0.498)	(0.029)	(0.064)	(0.067)
Observations	999	999	999	999	999
Pseudo R^2	0.066	0.062	0.042	0.043	0.038
χ^2	255.3	228.4	176.5	168.4	56.42

Exponentiated coefficients; p-values in parentheses

* $p < 0.10$, ** $p < 0.05$, *** $p < 0.01$

Table 16. Odds ratios and p-values (in parentheses) from an ordered logit regression interacting the treatment groups with information avoidance, cognitive load, credibility, warm glow, consistency and controlling for participants' demographics. The full regression tables can be found in the Appendix.

	(1) WTP CN	(2) WTP Net-Zero	(3) Tot Understanding	(4) Multiple Choice	(5) Open Question
Information	1	1	1	1	1
Bullet Points	8.485*	1.999	5.143**	4.565*	3.198
	(0.058)	(0.488)	(0.048)	(0.071)	(0.235)
Traffic Lights	2.914	0.268	0.560	0.631	0.707
	(0.388)	(0.240)	(0.472)	(0.575)	(0.708)
Warm Glow CN	3.283***	1.289	0.702**	0.683**	1.030
	(0.000)	(0.128)	(0.037)	(0.024)	(0.881)
Warm Glow Net-Zero	0.952	2.268***	1.343	1.290	1.266
	(0.820)	(0.000)	(0.127)	(0.189)	(0.260)
Bullet Points × Warm Glow CN	0.612*	0.724	1.552	1.689**	0.895
	(0.065)	(0.188)	(0.104)	(0.046)	(0.698)
Traffic Lights × Warm Glow CN	0.585*	0.843	1.643**	1.759**	0.866
	(0.058)	(0.468)	(0.050)	(0.024)	(0.576)
Bullet Points × Warm Glow Net-Zero	1.429	1.328	0.666	0.636	0.965
	(0.249)	(0.357)	(0.168)	(0.120)	(0.907)
Traffic Lights × Warm Glow Net-Zero	1.442	1.361	0.830	0.869	0.816
	(0.228)	(0.256)	(0.531)	(0.629)	(0.453)
Cognitive Load	1.098	0.982	0.904*	0.922	0.918
	(0.315)	(0.790)	(0.096)	(0.205)	(0.339)
Bullet Points × Cognitive Load	0.862	1.038	0.969	0.948	1.066
	(0.202)	(0.707)	(0.771)	(0.638)	(0.625)
Traffic Lights × Cognitive Load	0.952	1.238*	1.150	1.139	1.060
	(0.695)	(0.059)	(0.161)	(0.207)	(0.633)
Info Avoidance (PCA)	0.944	0.879*	1.063	1.038	1.121
	(0.493)	(0.094)	(0.429)	(0.657)	(0.193)
Bullet Points × Info Avoidance (PCA)	0.947	0.949	1.126	1.097	1.101
	(0.637)	(0.654)	(0.314)	(0.462)	(0.458)
Traffic Lights × Info Avoidance (PCA)	1.033	1.138	0.976	0.997	0.922
	(0.790)	(0.278)	(0.831)	(0.981)	(0.507)
Total Understanding	0.953	0.960			
	(0.395)	(0.446)			
Bullet Points × Total Understanding	1.034	1.070			
	(0.649)	(0.352)			
Traffic Lights × Total Understanding	1.001	1.008			
	(0.991)	(0.922)			
Bullet Points × Credibility CN	1.038	1.062	0.788	0.890	0.524***
	(0.841)	(0.735)	(0.102)	(0.434)	(0.001)
Information × Credibility CN	2.029***	1.508**	0.686**	0.704**	0.787
	(0.000)	(0.018)	(0.011)	(0.032)	(0.245)
Traffic Lights × Credibility CN	1.027	0.965	0.819	0.822	0.919
	(0.892)	(0.848)	(0.240)	(0.247)	(0.636)
Bullet Points × Credibility Net-Zero	1.027	0.931	0.810	0.825	0.899
	(0.879)	(0.681)	(0.217)	(0.268)	(0.591)
Information × Credibility Net-Zero	0.962	1.166	1.196	1.204	0.990
	(0.804)	(0.303)	(0.180)	(0.210)	(0.953)
Traffic Lights × Credibility Net-Zero	1.429	1.594**	0.794	0.731*	1.180
	(0.114)	(0.019)	(0.189)	(0.072)	(0.344)
Consistency	0.642***	1.162	1.254*	1.226	1.242
	(0.002)	(0.286)	(0.083)	(0.122)	(0.153)
Observations	750	750	750	750	750
Pseudo R^2	0.065	0.063	0.021	0.024	0.027
χ^2	177.3	161.4	62.52	63.62	32.29

Exponentiated coefficients; *p*-values in parentheses

* $p < 0.10$, ** $p < 0.05$, *** $p < 0.01$

Table 17. Odds ratios and p-values (in parentheses) from an ordered logit regression interacting the treatment groups with information avoidance, cognitive load, credibility, warm glow, consistency and controlling for participants' demographics excluding the control group. The full regression tables can be found in the Appendix.

	(1)	(2)	(3)	(4)	(5)	(6)	(7)	(8)
	WTP CN	WTP CN	WTP CN	WTP CN	WTP CN	WTP CN	WTP CN	WTP CN
Information	-0.726	-0.715	-0.491	-0.432	-0.302	-0.135	-0.0238	
	(0.110)	(0.115)	(0.317)	(0.380)	(0.539)	(0.785)	(0.965)	
Bullet Points	-0.835*	-0.823*	-0.352	-0.314	-0.735	-0.193	-0.380	
	(0.056)	(0.059)	(0.472)	(0.525)	(0.110)	(0.699)	(0.487)	
Traffic Lights	-0.571	-0.609	-0.0725	-0.0424	-0.651	-0.213	-0.239	
	(0.216)	(0.185)	(0.886)	(0.933)	(0.191)	(0.678)	(0.668)	
Education		-0.0377	-0.0507	-0.0539			-0.104	-0.100
		(0.506)	(0.410)	(0.380)			(0.124)	(0.140)
Age		0.0127	0.0270**	0.0260**			0.0231	0.0256*
		(0.222)	(0.014)	(0.018)			(0.101)	(0.072)
Female		-0.566*	-0.685**	-0.704**			-1.102***	-1.017***
		(0.076)	(0.042)	(0.037)			(0.003)	(0.007)
Earnings		0.0620	0.0821*	0.0827*				
		(0.183)	(0.096)	(0.094)				
Democrat		1.090***	0.228	0.240			-0.201	-0.178
		(0.001)	(0.512)	(0.491)			(0.690)	(0.725)
Understanding: visual			-0.370***					
			(0.000)					
Consistency			-1.231***	-1.209***			-1.053***	-1.107***
			(0.000)	(0.000)			(0.006)	(0.005)
Total Understanding				-0.352***		-0.254***	-0.223***	
				(0.000)		(0.001)	(0.009)	
Warm Glow Net-Zero					0.156	0.295	0.0888	0.566
					(0.631)	(0.365)	(0.797)	(0.414)
Warm Glow Carbon Neutral					1.934***	2.116***	2.021***	1.509**
					(0.000)	(0.000)	(0.000)	(0.022)
Cognitive Load					0.192*	0.181*	0.200*	-0.187
					(0.074)	(0.088)	(0.096)	(0.407)
Worry about Climate Change (PCA)					0.0492	0.196	0.193	
					(0.720)	(0.139)	(0.341)	
Info Avoidance (PCA)					-0.123	-0.0722	-0.107	-0.00995
					(0.302)	(0.543)	(0.429)	(0.969)
Credibility: Carbon Neutral					0.777***		0.812***	0.481
					(0.004)		(0.004)	(0.374)
Credibility: Net-Zero					0.204		0.167	0.293
					(0.428)		(0.540)	(0.610)
$10,000 to $19,999							-0.0632	0.335
							(0.953)	(0.738)
$20,000 to $29,999							1.450	1.874*
							(0.192)	(0.069)
$30,000 to $39,999							0.809	1.300
							(0.435)	(0.180)
$40,000 to $49,999							1.541	1.849*
							(0.167)	(0.069)
$50,000 to $59,999							1.395	1.801*
							(0.179)	(0.060)
$60,000 to $69,999							2.043*	2.507**
							(0.075)	(0.019)
$70,000 to $79,999							0.863	1.006
							(0.426)	(0.319)
$80,000 to $89,999							2.261*	2.751**
							(0.070)	(0.018)
$90,000 to $99,999							0.725	0.961
							(0.523)	(0.364)
$100,000 to $149,999							1.161	1.373
							(0.246)	(0.130)
$150,000 or more							0.564	0.855
							(0.578)	(0.360)
White							0.220	0.246
							(0.632)	(0.588)
Political Scale							0.0351	0.0707
							(0.787)	(0.585)
Employed							-0.164	-0.0764
							(0.713)	(0.864)
Retired							-1.047	-1.100
							(0.142)	(0.123)
Republican							0.194	-0.113
							(0.776)	(0.866)
Color Deficiency							2.061*	1.855
							(0.062)	(0.109)
Control								-2.092
								(0.356)
Information								-5.005**
								(0.040)
Traffic Lights								-2.504
								(0.308)
Control × Warm Glow Carbon Neutral								1.443
								(0.130)
Information × Warm Glow Carbon Neutral								1.254
								(0.130)
Traffic Lights × Warm Glow Carbon Neutral								-0.111
								(0.903)
Control × Warm Glow Net-Zero								-1.200
								(0.223)
Information × Warm Glow Net-Zero								-1.147
								(0.191)
Traffic Lights × Warm Glow Net-Zero								0.0507
								(0.958)
Control × Info Avoidance (PCA)								-0.358
								(0.350)
Information × Info Avoidance (PCA)								-0.129
								(0.715)
Traffic Lights × Info Avoidance (PCA)								0.185
								(0.601)
Control × Credibility: Carbon Neutral								0.312
								(0.701)
Information × Credibility: Carbon Neutral								1.324*
								(0.091)
Traffic Lights × Credibility: Carbon Neutral								-0.100
								(0.898)
Control × Credibility: Net-Zero								-0.621
								(0.425)
Information × Credibility: Net-Zero								0.172
								(0.818)
Traffic Lights × Credibility: Net-Zero								0.312
								(0.721)
Control × Cognitive Load								0.629*
								(0.060)
Information × Cognitive Load								0.436
								(0.193)
Traffic Lights × Cognitive Load								0.488
								(0.118)
Constant	7.027***	6.081***	8.373***	8.488***	-1.228	0.974	-0.789	-0.498
	(0.000)	(0.000)	(0.000)	(0.000)	(0.140)	(0.215)	(0.634)	(0.774)
Observations	1487	1486	1313	1313	1101	1102	990	992
R^2	0.003	0.014	0.038	0.039	0.196	0.191	0.195	0.209

p-values in parentheses

* $p < 0.10$, ** $p < 0.05$, *** $p < 0.01$

Table 18. Determinants of WTP for a company with a carbon neutral claim in Study II. OLS regression models with robust standard errors. Model 1: group assignment only. Model 2: group assignment plus basic demographics (qualification, age, gender, earnings, Democrat identification). Model 3: Model 2 plus consistency. Model 4: basic demographics, group assignment, and mechanisms (warm glow, cognitive load, information avoidance, climate change concern). Model 5: Model 4 plus consistency. Model 6: Model 5 plus credibility and interactions between the mechanisms and the treatment groups.

	(1)	(2)	(3)	(4)	(5)	(6)	(7)	(8)
	WTP NZ	WTP NZ	WTP NZ	WTP NZ	WTP NZ	WTP NZ	WTP NZ	WTP NZ
Information	-0.0853	-0.0514	0.197	0.211	-0.204	-0.141	-0.0155	
	(0.852)	(0.910)	(0.692)	(0.674)	(0.684)	(0.784)	(0.978)	
Bullet Points	-0.608	-0.576	-0.257	-0.279	-0.684	-0.392	-0.368	
	(0.174)	(0.195)	(0.617)	(0.588)	(0.149)	(0.452)	(0.529)	
Traffic Lights	0.578	0.538	1.023*	1.010*	0.138	0.392	0.401	
	(0.238)	(0.265)	(0.056)	(0.059)	(0.791)	(0.469)	(0.496)	
Education		-0.0301	-0.0371	-0.0388			-0.104	-0.100
		(0.604)	(0.549)	(0.531)			(0.124)	(0.141)
Age		0.0270**	0.0445***	0.0441***			0.0353**	0.0399***
		(0.012)	(0.000)	(0.000)			(0.018)	(0.008)
Female		-0.662**	-0.783**	-0.795**			-1.237***	-1.110***
		(0.044)	(0.025)	(0.023)			(0.001)	(0.005)
Earnings		0.115**	0.120**	0.120**				
		(0.016)	(0.018)	(0.017)				
Democrat		1.416***	0.526	0.528			0.0378	0.0199
		(0.000)	(0.140)	(0.139)			(0.937)	(0.967)
Understanding: visual			-0.219**					
			(0.018)					
Consistency			0.0754	0.0821			0.149	0.0798
			(0.830)	(0.815)			(0.702)	(0.841)
Total Understanding				-0.186**		-0.133	-0.115	
				(0.027)		(0.117)	(0.209)	
Warm Glow Net-Zero					2.133***	2.256***	2.083***	2.278***
					(0.000)	(0.000)	(0.000)	(0.001)
Warm Glow Carbon Neutral					0.192	0.353	0.213	-0.139
					(0.462)	(0.171)	(0.438)	(0.823)
Cognitive Load					0.236**	0.233**	0.248**	0.0482
					(0.031)	(0.035)	(0.042)	(0.819)
Worry about Climate Change (PCA)					0.0609	0.172	0.224	
					(0.669)	(0.211)	(0.312)	
Info Avoidance (PCA)					-0.156	-0.117	-0.168	-0.122
					(0.203)	(0.337)	(0.217)	(0.648)
Credibility: Carbon Neutral					0.629**		0.720**	0.503
					(0.024)		(0.014)	(0.350)
Credibility: Net-Zero					0.223		0.264	-0.0756
					(0.394)		(0.344)	(0.895)
$10,000 to $19,999							1.370	1.439
							(0.129)	(0.124)
$20,000 to $29,999							2.261**	2.380**
							(0.013)	(0.011)
$30,000 to $39,999							2.084**	2.235**
							(0.016)	(0.012)
$40,000 to $49,999							2.850***	2.891***
							(0.002)	(0.002)
$50,000 to $59,999							2.176**	2.244**
							(0.014)	(0.012)
$60,000 to $69,999							2.857***	3.039***
							(0.003)	(0.001)
$70,000 to $79,999							2.315**	2.317**
							(0.011)	(0.011)
$80,000 to $89,999							3.465***	3.622***
							(0.002)	(0.001)
$90,000 to $99,999							1.967**	2.050**
							(0.045)	(0.033)
$100,000 to $149,999							2.038**	1.994**
							(0.012)	(0.015)
$150,000 or more							1.907**	1.971**
							(0.031)	(0.026)
White							-0.0875	-0.0452
							(0.852)	(0.922)
Political Scale							0.0450	0.0349
							(0.730)	(0.782)
Employed							0.0101	0.0222
							(0.983)	(0.962)
Retired							-0.601	-0.721
							(0.439)	(0.357)
Republican							0.658	0.432
							(0.333)	(0.521)
Color Deficiency							3.249***	3.235**
							(0.010)	(0.013)
Control								-1.611
								(0.461)
Information								-1.962
								(0.400)
Traffic Lights								-6.539***
								(0.006)
Control × Warm Glow Carbon Neutral								0.623
								(0.467)
Information × Warm Glow Carbon Neutral								0.676
								(0.397)
Traffic Lights × Warm Glow Carbon Neutral								0.294
								(0.714)
Control × Warm Glow Net-Zero								-0.197
								(0.835)
Information × Warm Glow Net-Zero								-0.650
								(0.475)
Traffic Lights × Warm Glow Net-Zero								0.206
								(0.804)
Control × Info Avoidance (PCA)								-0.182
								(0.644)
Information × Info Avoidance (PCA)								-0.210
								(0.566)
Traffic Lights × Info Avoidance (PCA)								0.291
								(0.442)
Control × Credibility: Carbon Neutral								0.372
								(0.662)
Information × Credibility: Carbon Neutral								0.961
								(0.211)
Traffic Lights × Credibility: Carbon Neutral								-0.186
								(0.821)
Control × Credibility: Net-Zero								-0.273
								(0.737)
Information × Credibility: Net-Zero								0.536
								(0.463)
Traffic Lights × Credibility: Net-Zero								1.300
								(0.121)
Control × Cognitive Load								0.194
								(0.537)
Information × Cognitive Load								-0.138
								(0.654)
Traffic Lights × Cognitive Load								0.669**
								(0.044)
Constant	7.176***	5.057***	5.989***	5.986***	-1.455*	0.123	-4.162***	-2.690
	(0.000)	(0.000)	(0.000)	(0.000)	(0.084)	(0.877)	(0.005)	(0.104)
Observations	1487	1486	1313	1313	1101	1102	990	992
R^2	0.004	0.026	0.033	0.033	0.207	0.200	0.200	0.214

p-values in parentheses

* $p < 0.10$, ** $p < 0.05$, *** $p < 0.01$

Table 19. Determinants of WTP for a company with a net-zero claim in Study II. OLS regression models with robust standard errors. Model 1: group assignment only. Model 2: group assignment plus basic demographics (qualification, age, gender, earnings, Democrat identification). Model 3: Model 2 plus consistency. Model 4: basic demographics, group assignment, and mechanisms (warm glow, cognitive load, information avoidance, climate change concern). Model 5: Model 4 plus consistency. Model 6: Model 5 plus credibility and interactions between the mechanisms and the treatment groups.

	(1)	(2)	(3)	(4)	(5)	(6)	(7)	(8)
	Δ WTP	Δ WTP	Δ WTP	Δ WTP	Δ WTP	Δ WTP	Δ WTP	Δ WTP
Information	0.640**	0.663**	0.688**	0.643**	0.0982	-0.00561	0.00827	
	(0.017)	(0.014)	(0.024)	(0.034)	(0.747)	(0.985)	(0.981)	
Bullet Points	0.227	0.247	0.0958	0.0345	0.0504	-0.199	0.0124	
	(0.452)	(0.413)	(0.796)	(0.926)	(0.875)	(0.584)	(0.976)	
Traffic Lights	1.148***	1.146***	1.096***	1.053***	0.790**	0.604*	0.641*	
	(0.000)	(0.000)	(0.001)	(0.001)	(0.022)	(0.079)	(0.097)	
Education		0.00761	0.0136	0.0151			0.000703	0.000133
		(0.837)	(0.743)	(0.715)			(0.987)	(0.998)
Age		0.0143**	0.0175**	0.0180**			0.0122	0.0143
		(0.041)	(0.022)	(0.018)			(0.211)	(0.144)
Female		-0.0962	-0.0977	-0.0910			-0.135	-0.0926
		(0.644)	(0.664)	(0.686)			(0.595)	(0.718)
Earnings		0.0529*	0.0378	0.0375				
		(0.091)	(0.266)	(0.271)				
Democrat		0.325	0.298	0.288			0.239	0.198
		(0.122)	(0.202)	(0.217)			(0.471)	(0.535)
Understanding: visual			0.152**					
			(0.017)					
Consistency			1.307***	1.291***			1.202***	1.186***
			(0.000)	(0.000)			(0.000)	(0.000)
Total Understanding				0.166***		0.120**	0.108*	
				(0.004)		(0.034)	(0.079)	
Warm Glow Net-Zero					1.977***	1.961***	1.994***	1.713***
					(0.000)	(0.000)	(0.000)	(0.000)
Warm Glow Carbon Neutral					-1.742***	-1.763***	-1.808***	-1.649***
					(0.000)	(0.000)	(0.000)	(0.000)
Cognitive Load					0.0445	0.0525	0.0482	0.236
					(0.612)	(0.545)	(0.617)	(0.120)
Worry about Climate Change (PCA)					0.0117	-0.0245	0.0309	
					(0.902)	(0.780)	(0.817)	
Info Avoidance (PCA)					-0.0335	-0.0451	-0.0613	-0.112
					(0.695)	(0.600)	(0.519)	(0.461)
Credibility: Carbon Neutral					-0.148		-0.0927	0.0220
					(0.411)		(0.625)	(0.948)
Credibility: Net-Zero					0.0185		0.0971	-0.369
					(0.914)		(0.581)	(0.303)
$10,000 to $19,999							1.433	1.104
							(0.130)	(0.170)
$20,000 to $29,999							0.811	0.506
							(0.410)	(0.562)
$30,000 to $39,999							1.276	0.936
							(0.161)	(0.233)
$40,000 to $49,999							1.309	1.043
							(0.200)	(0.243)
$50,000 to $59,999							0.781	0.443
							(0.371)	(0.560)
$60,000 to $69,999							0.814	0.533
							(0.402)	(0.534)
$70,000 to $79,999							1.452	1.311*
							(0.112)	(0.096)
$80,000 to $89,999							1.204	0.870
							(0.213)	(0.301)
$90,000 to $99,999							1.242	1.088
							(0.239)	(0.243)
$100,000 to $149,999							0.877	0.621
							(0.320)	(0.410)
$150,000 or more							1.342	1.116
							(0.138)	(0.161)
White							-0.307	-0.291
							(0.392)	(0.409)
Political Scale							0.00998	-0.0358
							(0.924)	(0.725)
Employed							0.174	0.0986
							(0.585)	(0.752)
Retired							0.447	0.380
							(0.348)	(0.439)
Republican							0.464	0.545
							(0.329)	(0.242)
Color Deficiency							1.188	1.380
							(0.213)	(0.163)
Control								0.481
								(0.760)
Information								3.043**
								(0.042)
Traffic Lights								-4.036**
								(0.047)
Control × Warm Glow Carbon Neutral								-0.820
								(0.272)
Information × Warm Glow Carbon Neutral								-0.578
								(0.311)
Traffic Lights × Warm Glow Carbon Neutral								0.406
								(0.520)
Control × Warm Glow Net-Zero								1.003
								(0.216)
Information × Warm Glow Net-Zero								0.497
								(0.420)
Traffic Lights × Warm Glow Net-Zero								0.155
								(0.832)
Control × Info Avoidance (PCA)								0.176
								(0.477)
Information × Info Avoidance (PCA)								-0.0812
								(0.706)
Traffic Lights × Info Avoidance (PCA)								0.105
								(0.674)
Control × Credibility: Carbon Neutral								0.0601
								(0.912)
Information × Credibility: Carbon Neutral								-0.363
								(0.445)
Traffic Lights × Credibility: Carbon Neutral								-0.0859
								(0.869)
Control × Credibility: Net-Zero								0.347
								(0.490)
Information × Credibility: Net-Zero								0.364
								(0.433)
Traffic Lights × Credibility: Net-Zero								0.988*
								(0.074)
Control × Cognitive Load								-0.435*
								(0.073)
Information × Cognitive Load								-0.574***
								(0.006)
Traffic Lights × Cognitive Load								0.181
								(0.504)
Constant	0.149	-1.024*	-2.384***	-2.503***	-0.227	-0.851	-3.373***	-2.193
	(0.460)	(0.085)	(0.000)	(0.000)	(0.700)	(0.110)	(0.010)	(0.121)
Observations	1487	1486	1313	1313	1101	1102	990	992
R^2	0.012	0.019	0.051	0.053	0.126	0.129	0.175	0.203

p-values in parentheses

* $p < 0.10$, ** $p < 0.05$, *** $p < 0.01$

Table 20. Determinants of the difference in WTP for a company with a net-zero claim vs a carbon neutral claim in Study II. OLS regression models with robust standard errors. Model 1: group assignment only. Model 2: group assignment plus basic demographics (qualification, age, gender, earnings, Democrat identification). Model 3: Model 2 plus consistency. Model 4: basic demographics, group assignment, and mechanisms (warm glow, cognitive load, information avoidance, climate change concern). Model 5: Model 4 plus consistency. Model 6: Model 5 plus credibility and interactions between the mechanisms and the treatment groups.

	(1)	(2)	(3)	(4)	(5)	(6)	(7)	(8)
	WTP CN	WTP CN	WTP CN	WTP CN	WTP CN	WTP CN	WTP CN	WTP CN
Bullet Points	-0.109	-0.110	0.0875	0.0630	-0.454	-0.0829	-0.289	
	(0.802)	(0.802)	(0.853)	(0.894)	(0.328)	(0.865)	(0.584)	
Traffic Lights	0.155	0.105	0.418	0.388	-0.360	-0.0897	-0.00960	
	(0.736)	(0.819)	(0.396)	(0.430)	(0.472)	(0.858)	(0.986)	
Education		-0.00724	-0.0307	-0.0338			-0.107	-0.110
		(0.910)	(0.660)	(0.627)			(0.174)	(0.163)
Age		0.0136	0.0239*	0.0227*			0.0156	0.0232
		(0.264)	(0.059)	(0.073)			(0.351)	(0.169)
Female		-0.518	-0.899**	-0.913**			-1.365***	-1.219***
		(0.158)	(0.021)	(0.019)			(0.002)	(0.006)
Earnings		0.0592	0.102*	0.103*				
		(0.259)	(0.065)	(0.065)				
Democrat		1.032***	0.107	0.127			-0.434	-0.509
		(0.005)	(0.788)	(0.750)			(0.467)	(0.391)
Understanding: visual			-0.362***					
			(0.000)					
Consistency			-1.578***	-1.559***			-1.308***	-1.251***
			(0.000)	(0.000)			(0.003)	(0.005)
Total Understanding				-0.339***		-0.248***	-0.234**	
				(0.000)		(0.004)	(0.011)	
Warm Glow Net-Zero					0.284	0.472	0.437	0.525
					(0.426)	(0.187)	(0.248)	(0.455)
Warm Glow Carbon Neutral					1.714***	1.897***	1.956***	1.561**
					(0.000)	(0.000)	(0.000)	(0.019)
Cognitive Load					0.102	0.0963	0.0862	-0.181
					(0.405)	(0.434)	(0.531)	(0.423)
Worry about Climate Change (PCA)					0.00617	0.182	0.235	
					(0.968)	(0.229)	(0.315)	
Info Avoidance (PCA)					0.0155	0.0590	0.0878	0.0318
					(0.906)	(0.654)	(0.558)	(0.901)
Credibility: Carbon Neutral					0.810***			0.483
					(0.009)			(0.376)
Credibility: Net-Zero					0.333			0.345
					(0.273)			(0.555)
$10,000 to $19,999							0.634	0.619
							(0.541)	(0.555)
$20,000 to $29,999							1.043	1.434
							(0.355)	(0.207)
$30,000 to $39,999							1.287	1.626
							(0.200)	(0.112)
$40,000 to $49,999							1.814	1.861*
							(0.104)	(0.090)
$50,000 to $59,999							1.573	1.758*
							(0.126)	(0.091)
$60,000 to $69,999							1.698	2.052*
							(0.136)	(0.072)
$70,000 to $79,999							0.895	0.832
							(0.396)	(0.432)
$80,000 to $89,999							2.684**	2.863**
							(0.031)	(0.020)
$90,000 to $99,999							1.678	1.709
							(0.156)	(0.147)
$100,000 to $149,999							1.852*	1.748*
							(0.062)	(0.074)
$150,000 or more							0.641	0.769
							(0.504)	(0.425)
White							0.0917	0.110
							(0.867)	(0.838)
Political Scale							0.0941	0.136
							(0.545)	(0.378)
Employed							-0.198	-0.181
							(0.701)	(0.724)
Retired							-0.991	-1.116
							(0.215)	(0.164)
Republican							0.209	-0.229
							(0.792)	(0.765)
Color Deficiency							2.646*	2.419*
							(0.054)	(0.091)
Information								-4.683*
								(0.057)
Traffic Lights								-2.032
								(0.406)
Information × Warm Glow Carbon Neutral								1.160
								(0.162)
Traffic Lights × Warm Glow Carbon Neutral								-0.186
								(0.838)
Information × Warm Glow Net-Zero								-1.030
								(0.243)
Traffic Lights × Warm Glow Net-Zero								0.153
								(0.874)
Information × Info Avoidance (PCA)								-0.163
								(0.647)
Traffic Lights × Info Avoidance (PCA)								0.159
								(0.654)
Information × Credibility: Carbon Neutral								1.244
								(0.115)
Traffic Lights × Credibility: Carbon Neutral								-0.218
								(0.783)
Information × Credibility: Net-Zero								0.164
								(0.828)
Traffic Lights × Credibility: Net-Zero								0.354
								(0.689)
Information × Cognitive Load								0.397
								(0.234)
Traffic Lights × Cognitive Load								0.423
								(0.176)
Constant	6.301***	5.107***	8.093***	8.248***	-1.206	1.344	1.589	-0.202
	(0.000)	(0.000)	(0.000)	(0.000)	(0.234)	(0.184)	(0.368)	(0.911)
Observations	1117	1117	990	990	830	831	743	744
R^2	0.000	0.010	0.045	0.046	0.185	0.177	0.185	0.209

p-values in parentheses
* $p < 0.10$, ** $p < 0.05$, *** $p < 0.01$

Table 21. Determinants of WTP for a company with a carbon neutral claim in Study II. OLS regression models with robust standard errors excluding the control group. Model 1: group assignment only. Model 2: group assignment plus basic demographics (qualification, age, gender, earnings, Democrat identification). Model 3: Model 2 plus consistency. Model 4: basic demographics, group assignment, and mechanisms (warm glow, cognitive load, information avoidance, climate change concern). Model 5: Model 4 plus consistency. Model 6: Model 5 plus credibility and interactions between the mechanisms and the treatment groups.

	(1)	(2)	(3)	(4)	(5)	(6)	(7)	(8)
	WTP NZ	WTP NZ	WTP NZ	WTP NZ	WTP NZ	WTP NZ	WTP NZ	WTP NZ
Bullet Points	-0.522	-0.520	-0.404	-0.442	-0.457	-0.220	-0.201	
	(0.234)	(0.238)	(0.400)	(0.356)	(0.333)	(0.654)	(0.708)	
Traffic Lights	0.663	0.585	0.841	0.812	0.371	0.554	0.624	
	(0.170)	(0.222)	(0.102)	(0.114)	(0.473)	(0.285)	(0.268)	
Education		0.00603	-0.0120	-0.0139			-0.0810	-0.0798
		(0.928)	(0.867)	(0.846)			(0.298)	(0.308)
Age		0.0291**	0.0425***	0.0418***			0.0321*	0.0414**
		(0.020)	(0.001)	(0.002)			(0.062)	(0.017)
Female		-0.507	-0.841**	-0.851**			-1.459***	-1.231***
		(0.183)	(0.038)	(0.036)			(0.001)	(0.007)
Earnings		0.0898	0.109*	0.109*				
		(0.103)	(0.059)	(0.059)				
Democrat		1.414***	0.462	0.471			-0.465	-0.623
		(0.000)	(0.264)	(0.254)			(0.410)	(0.268)
Understanding: visual			-0.262***					
			(0.009)					
Consistency			0.244	0.252			0.342	0.343
			(0.542)	(0.530)			(0.441)	(0.441)
Total Understanding				-0.227**		-0.149	-0.169*	
				(0.013)		(0.106)	(0.090)	
Warm Glow Net-Zero					2.109***	2.286***	2.271***	2.238***
					(0.000)	(0.000)	(0.000)	(0.001)
Warm Glow Carbon Neutral					0.117	0.262	0.314	-0.0960
					(0.691)	(0.360)	(0.309)	(0.876)
Cognitive Load					0.248*	0.246*	0.241*	0.0611
					(0.051)	(0.057)	(0.096)	(0.772)
Worry about Climate Change (PCA)					0.0286	0.170	0.276	
					(0.864)	(0.291)	(0.291)	
Info Avoidance (PCA)					-0.0638	-0.0309	-0.0474	-0.0937
					(0.647)	(0.825)	(0.762)	(0.726)
Credibility: Carbon Neutral					0.588*			0.630
					(0.062)			(0.239)
Credibility: Net-Zero					0.390			-0.0904
					(0.196)			(0.877)
$10,000 to $19,999							0.972	0.900
							(0.360)	(0.405)
$20,000 to $29,999							0.957	1.178
							(0.393)	(0.305)
$30,000 to $39,999							1.606	1.786*
							(0.131)	(0.097)
$40,000 to $49,999							2.305**	2.308**
							(0.047)	(0.047)
$50,000 to $59,999							1.545	1.567
							(0.150)	(0.153)
$60,000 to $69,999							1.227	1.414
							(0.264)	(0.192)
$70,000 to $79,999							1.641	1.586
							(0.125)	(0.138)
$80,000 to $89,999							2.778**	2.838**
							(0.033)	(0.023)
$90,000 to $99,999							2.518*	2.550**
							(0.052)	(0.042)
$100,000 to $149,999							1.514	1.322
							(0.131)	(0.180)
$150,000 or more							0.923	1.003
							(0.358)	(0.321)
White							-0.277	-0.204
							(0.622)	(0.714)
Political Scale							-0.0393	-0.0429
							(0.804)	(0.777)
Employed							-0.0284	-0.109
							(0.958)	(0.837)
Retired							-0.433	-0.648
							(0.620)	(0.458)
Republican							0.662	0.302
							(0.405)	(0.695)
Color Deficiency							4.186***	4.179***
							(0.005)	(0.007)
Information								-1.499
								(0.528)
Traffic Lights								-6.296***
								(0.008)
Information × Warm Glow Carbon Neutral								0.643
								(0.415)
Traffic Lights × Warm Glow Carbon Neutral								0.247
								(0.757)
Information × Warm Glow Net-Zero								-0.583
								(0.520)
Traffic Lights × Warm Glow Net-Zero								0.306
								(0.713)
Information × Info Avoidance (PCA)								-0.234
								(0.527)
Traffic Lights × Info Avoidance (PCA)								0.250
								(0.507)
Information × Credibility: Carbon Neutral								0.780
								(0.312)
Traffic Lights × Credibility: Carbon Neutral								-0.446
								(0.593)
Information × Credibility: Net-Zero								0.547
								(0.458)
Traffic Lights × Credibility: Net-Zero								1.449*
								(0.087)
Information × Cognitive Load								-0.176
								(0.570)
Traffic Lights × Cognitive Load								0.640*
								(0.054)
Constant	7.090***	4.692***	6.279***	6.308***	-1.780*	0.124	-1.272	-1.830
	(0.000)	(0.000)	(0.000)	(0.000)	(0.082)	(0.904)	(0.455)	(0.304)
Observations	1117	1117	990	990	830	831	743	744
R^2	0.006	0.025	0.035	0.035	0.205	0.197	0.197	0.226

p-values in parentheses

* $p < 0.10$, ** $p < 0.05$, *** $p < 0.01$

Table 22. Determinants of WTP for a company with a net-zero claim in Study II. OLS regression models with robust standard errors excluding the control group. Model 1: group assignment only. Model 2: group assignment plus basic demographics (qualification, age, gender, earnings, Democrat identification). Model 3: Model 2 plus consistency. Model 4: basic demographics, group assignment, and mechanisms (warm glow, cognitive load, information avoidance, climate change concern). Model 5: Model 4 plus consistency. Model 6: Model 5 plus credibility and interactions between the mechanisms and the treatment groups.

	(1)	(2)	(3)	(4)	(5)	(6)	(7)	(8)
	Δ WTP	Δ WTP	Δ WTP	Δ WTP	Δ WTP	Δ WTP	Δ WTP	Δ WTP
Bullet Points	-0.413	-0.411	-0.491	-0.505	-0.00320	-0.137	0.0874	
	(0.151)	(0.154)	(0.141)	(0.128)	(0.992)	(0.688)	(0.809)	
Traffic Lights	0.508*	0.480*	0.424	0.424	0.731**	0.644**	0.634*	
	(0.068)	(0.085)	(0.162)	(0.162)	(0.021)	(0.044)	(0.067)	
Education		0.0133	0.0187	0.0199			0.0260	0.0298
		(0.761)	(0.695)	(0.675)			(0.603)	(0.560)
Age		0.0155*	0.0186**	0.0190**			0.0165	0.0182
		(0.073)	(0.045)	(0.039)			(0.153)	(0.121)
Female		0.0109	0.0584	0.0622			-0.0946	-0.0113
		(0.964)	(0.819)	(0.808)			(0.745)	(0.970)
Earnings		0.0306	0.00629	0.00635				
		(0.371)	(0.862)	(0.861)				
Democrat		0.383	0.354	0.344			-0.0302	-0.114
		(0.117)	(0.183)	(0.196)			(0.937)	(0.755)
Understanding: visual			0.100					
			(0.141)					
Consistency			1.823***	1.810***			1.650***	1.594***
			(0.000)	(0.000)			(0.000)	(0.000)
Total Understanding				0.112*		0.0986	0.0655	
				(0.064)		(0.104)	(0.304)	
Warm Glow Net-Zero					1.825***	1.814***	1.833***	1.713***
					(0.000)	(0.000)	(0.000)	(0.000)
Warm Glow Carbon Neutral					-1.597***	-1.635***	-1.642***	-1.657***
					(0.000)	(0.000)	(0.000)	(0.000)
Cognitive Load					0.145	0.149	0.155	0.242
					(0.129)	(0.119)	(0.142)	(0.113)
Worry about Climate Change (PCA)					0.0224	-0.0119	0.0415	
					(0.830)	(0.904)	(0.774)	
Info Avoidance (PCA)					-0.0793	-0.0899	-0.135	-0.126
					(0.402)	(0.343)	(0.184)	(0.409)
Credibility: Carbon Neutral					-0.223			0.148
					(0.270)			(0.659)
Credibility: Net-Zero					0.0564			-0.436
					(0.774)			(0.230)
$10,000 to $19,999							0.337	0.281
							(0.632)	(0.673)
$20,000 to $29,999							-0.0862	-0.257
							(0.919)	(0.761)
$30,000 to $39,999							0.319	0.159
							(0.612)	(0.787)
$40,000 to $49,999							0.491	0.446
							(0.561)	(0.573)
$50,000 to $59,999							-0.0281	-0.191
							(0.962)	(0.733)
$60,000 to $69,999							-0.471	-0.638
							(0.555)	(0.402)
$70,000 to $79,999							0.745	0.754
							(0.268)	(0.214)
$80,000 to $89,999							0.0934	-0.0250
							(0.898)	(0.972)
$90,000 to $99,999							0.841	0.841
							(0.376)	(0.333)
$100,000 to $149,999							-0.338	-0.426
							(0.576)	(0.453)
$150,000 or more							0.282	0.234
							(0.651)	(0.687)
White							-0.369	-0.314
							(0.364)	(0.435)
Political Scale							-0.133	-0.179
							(0.263)	(0.122)
Employed							0.169	0.0719
							(0.643)	(0.841)
Retired							0.558	0.468
							(0.295)	(0.407)
Republican							0.452	0.531
							(0.403)	(0.330)
Color Deficiency							1.540	1.760
							(0.147)	(0.115)
Information								3.184**
								(0.035)
Traffic Lights								-4.263**
								(0.038)
Information × Warm Glow Carbon Neutral								-0.516
								(0.360)
Traffic Lights × Warm Glow Carbon Neutral								0.434
								(0.492)
Information × Warm Glow Net-Zero								0.447
								(0.469)
Traffic Lights × Warm Glow Net-Zero								0.153
								(0.837)
Information × Info Avoidance (PCA)								-0.0708
								(0.745)
Traffic Lights × Info Avoidance (PCA)								0.0906
								(0.712)
Information × Credibility: Carbon Neutral								-0.464
								(0.325)
Traffic Lights × Credibility: Carbon Neutral								-0.228
								(0.663)
Information × Credibility: Net-Zero								0.383
								(0.414)
Traffic Lights × Credibility: Net-Zero								1.095*
								(0.053)
Information × Cognitive Load								-0.573***
								(0.007)
Traffic Lights × Cognitive Load								0.217
								(0.420)
Constant	0.789***	-0.414	-1.814**	-1.940***	-0.574	-1.221*	-2.861**	-1.627
	(0.000)	(0.536)	(0.014)	(0.008)	(0.446)	(0.081)	(0.020)	(0.242)
Observations	1117	1117	990	990	830	831	743	744
R^2	0.009	0.015	0.069	0.070	0.119	0.120	0.191	0.224

p-values in parentheses
* $p < 0.10$, ** $p < 0.05$, *** $p < 0.01$

Table 23. Determinants of the difference in WTP for a company with a net-zero claim vs a carbon neutral claim in Study II. OLS regression models with robust standard errors excluding the control group. Model 1: group assignment only. Model 2: group assignment plus basic demographics (qualification, age, gender, earnings, Democrat identification). Model 3: Model 2 plus consistency. Model 4: basic demographics, group assignment, and mechanisms (warm glow, cognitive load, information avoidance, climate change concern). Model 5: Model 4 plus consistency. Model 6: Model 5 plus credibility and interactions between the mechanisms and the treatment groups.

	(1)	(2)	(3)	(4)	(5)	(6)	(7)
	Open	Open	Open	Open	Open	Open	Open
Open							
Information	0.689***	0.688***	0.785***	0.655***	0.751***	0.809***	
	(0.000)	(0.000)	(0.000)	(0.000)	(0.000)	(0.000)	
Bullet Points	0.653***	0.662***	0.751***	0.641***	0.747***	0.790***	
	(0.000)	(0.000)	(0.000)	(0.000)	(0.000)	(0.000)	
Traffic Lights	0.522***	0.532***	0.559***	0.505***	0.534***	0.582***	
	(0.000)	(0.000)	(0.000)	(0.004)	(0.003)	(0.002)	
Education		-0.0181	-0.0323			-0.0660***	-0.0642***
		(0.334)	(0.109)			(0.005)	(0.007)
Age		-0.00961***	-0.0106***			-0.00808*	-0.00803*
		(0.004)	(0.003)			(0.091)	(0.094)
Female		-0.149	-0.193*			-0.201	-0.178
		(0.163)	(0.091)			(0.137)	(0.198)
Earnings		0.0243	0.00773				
		(0.126)	(0.645)				
Democrat		0.202*	0.174			0.0207	0.0602
		(0.060)	(0.132)			(0.908)	(0.743)
Consistency			0.294**		0.268**	0.262*	0.238*
			(0.011)		(0.043)	(0.055)	(0.087)
Warm Glow Net-Zero				0.112	0.0915	0.164	0.238
				(0.236)	(0.347)	(0.118)	(0.309)
Warm Glow Carbon Neutral				-0.186*	-0.173*	-0.125	-0.0661
				(0.052)	(0.079)	(0.223)	(0.764)
Cognitive Load				-0.0597	-0.0527	-0.0427	-0.0101
				(0.146)	(0.226)	(0.341)	(0.917)
Worry about Climate Change (PCA)				0.0961*	0.131**	0.110	
				(0.060)	(0.035)	(0.126)	
Info Avoidance (PCA)				0.0858**	0.0805*	0.0865*	0.216**
				(0.046)	(0.077)	(0.065)	(0.027)
Less than $10,000						0	0
						(.)	(.)
$10,000 to $19,999						0.612	0.538
						(0.185)	(0.260)
$20,000 to $29,999						0.821*	0.887*
						(0.075)	(0.059)
$30,000 to $39,999						0.568	0.654
						(0.197)	(0.147)
$40,000 to $49,999						0.542	0.658
						(0.224)	(0.147)
$50,000 to $59,999						0.469	0.508
						(0.296)	(0.261)
$60,000 to $69,999						0.936**	1.046**
						(0.045)	(0.030)
$70,000 to $79,999						0.593	0.623
						(0.199)	(0.186)
$80,000 to $89,999						0.650	0.653
						(0.182)	(0.186)
$90,000 to $99,999						0.709	0.806*
						(0.137)	(0.095)
$100,000 to $149,999						0.771*	0.853*
						(0.076)	(0.054)
$150,000 or more						0.673	0.731
						(0.140)	(0.115)
White						0.119	0.144
						(0.482)	(0.404)
Political Scale						-0.0366	-0.0458
						(0.413)	(0.302)
Employed						-0.128	-0.150
						(0.506)	(0.450)
Retired						-0.262	-0.236
						(0.354)	(0.418)
Republican						0.0819	0.0278
						(0.727)	(0.905)
Color Deficiency						-0.487	-0.540
						(0.203)	(0.171)
Credibility: Carbon Neutral						-0.259**	-0.652***
						(0.012)	(0.002)
Credibility: Net-Zero						0.0428	-0.141
						(0.649)	(0.503)
Control							-2.176**
							(0.033)
Information							-1.430
							(0.161)
Traffic Lights							-1.275
							(0.205)
Control × Warm Glow Carbon Neutral							-0.312
							(0.354)
Information × Warm Glow Carbon Neutral							0.0558
							(0.854)
Traffic Lights × Warm Glow Carbon Neutral							-0.0423
							(0.879)
Control × Warm Glow Net-Zero							-0.00806
							(0.980)
Information × Warm Glow Net-Zero							0.147
							(0.652)
Traffic Lights × Warm Glow Net-Zero							-0.203
							(0.483)
Control × Info Avoidance (PCA)							-0.184
							(0.195)
Information × Info Avoidance (PCA)							-0.116
							(0.395)
Traffic Lights × Info Avoidance (PCA)							-0.181
							(0.172)
Control × Credibility: Carbon Neutral							0.659**
							(0.030)
Information × Credibility: Carbon Neutral							0.388
							(0.194)
Traffic Lights × Credibility: Carbon Neutral							0.595**
							(0.031)
Control × Credibility: Net-Zero							0.332
							(0.230)
Information × Credibility: Net-Zero							0.135
							(0.632)
Traffic Lights × Credibility: Net-Zero							0.248
							(0.362)
Control × Cognitive Load							-0.000804
							(0.995)
Information × Cognitive Load							-0.0483
							(0.723)
Traffic Lights × Cognitive Load							-0.0341
							(0.795)
Observations	1487	1486	1313	1102	998	990	992
Pseudo R^2	0.012	0.019	0.025	0.019	0.025	0.046	0.055
chi2	26.62	40.91	46.62	30.43	36.69	65.58	77.82

p-values in parentheses
* $p < 0.10$, ** $p < 0.05$, *** $p < 0.01$

Table 24. Determinants of correct answers in the open-ended question in Study II. Ordered logistic regression models with robust standard errors. Model 1: group assignment only. Model 2: group assignment plus basic demographics (qualification, age, gender, earnings, Democrat identification). Model 3: Model 2 plus consistency. Model 4: basic demographics, group assignment, and mechanisms (warm glow, cognitive load, information avoidance, climate change concern). Model 5: Model 4 plus consistency. Model 6: Model 5 plus credibility and interactions between the mechanisms and the treatment groups.

	(1)	(2)	(3)	(4)	(5)	(6)	(7)
	Visual U	Visual U	Visual U	Visual U	Visual U	Visual U	Visual U
Visual U							
Information	0.626***	0.603***	0.690***	0.692***	0.779***	0.764***	
	(0.000)	(0.000)	(0.000)	(0.000)	(0.000)	(0.000)	
Bullet Points	1.576***	1.577***	1.714***	1.715***	1.846***	1.872***	
	(0.000)	(0.000)	(0.000)	(0.000)	(0.000)	(0.000)	
Traffic Lights	1.054***	1.045***	1.095***	1.174***	1.210***	1.275***	
	(0.000)	(0.000)	(0.000)	(0.000)	(0.000)	(0.000)	
Education		0.00294	0.00444			0.0000221	0.00180
		(0.853)	(0.796)			(0.999)	(0.931)
Age		-0.000938	-0.00249			-0.00539	-0.00536
		(0.741)	(0.412)			(0.210)	(0.229)
Female		0.135	0.107			0.0812	0.0887
		(0.152)	(0.289)			(0.509)	(0.474)
Earnings		0.00670	0.00458				
		(0.620)	(0.753)				
Democrat		0.176*	0.144			-0.168	-0.159
		(0.061)	(0.153)			(0.290)	(0.326)
Consistency			0.191*		0.256**	0.228*	0.185
			(0.054)		(0.024)	(0.052)	(0.122)
Warm Glow Net-Zero				0.0111	0.0224	0.0677	-0.223
				(0.913)	(0.830)	(0.524)	(0.352)
Warm Glow Carbon Neutral				-0.0798	-0.110	-0.0883	0.163
				(0.409)	(0.260)	(0.365)	(0.454)
Cognitive Load				-0.0779**	-0.0742*	-0.0663*	-0.145
				(0.033)	(0.054)	(0.094)	(0.156)
Worry about Climate Change (PCA)				0.211***	0.189***	0.154**	
				(0.000)	(0.000)	(0.012)	
Info Avoidance (PCA)				0.0352	0.0196	0.0301	0.129
				(0.370)	(0.641)	(0.496)	(0.184)
$10,000 to $19,999						0.0688	0.147
						(0.866)	(0.713)
$20,000 to $29,999						0.429	0.340
						(0.300)	(0.404)
$30,000 to $39,999						0.178	0.136
						(0.640)	(0.713)
$40,000 to $49,999						0.183	0.341
						(0.629)	(0.357)
$50,000 to $59,999						-0.300	-0.335
						(0.435)	(0.366)
$60,000 to $69,999						0.311	0.342
						(0.439)	(0.385)
$70,000 to $79,999						0.589	0.593
						(0.134)	(0.122)
$80,000 to $89,999						0.227	0.183
						(0.610)	(0.680)
$90,000 to $99,999						0.193	0.240
						(0.649)	(0.563)
$100,000 to $149,999						0.559	0.608*
						(0.136)	(0.097)
$150,000 or more						0.482	0.470
						(0.235)	(0.234)
White						0.0408	0.0261
						(0.780)	(0.858)
Political Scale						-0.0732**	-0.0987***
						(0.043)	(0.007)
Employed						-0.440***	-0.504***
						(0.009)	(0.003)
Retired						0.0733	-0.0481
						(0.771)	(0.849)
Republican						-0.139	-0.177
						(0.494)	(0.390)
Color Deficiency						0.150	0.309
						(0.582)	(0.272)
Credibility: Carbon Neutral						-0.0539	-0.0765
						(0.539)	(0.642)
Credibility: Net-Zero						-0.107	-0.328*
						(0.205)	(0.091)
Control							-4.642***
							(0.000)
Information							-1.935**
							(0.040)
Traffic Lights							-2.228**
							(0.024)
Control × Warm Glow Carbon Neutral							-0.270
							(0.344)
Information × Warm Glow Carbon Neutral							-0.629**
							(0.030)
Traffic Lights × Warm Glow Carbon Neutral							-0.0416
							(0.881)
Control × Warm Glow Net-Zero							0.455
							(0.115)
Information × Warm Glow Net-Zero							0.493
							(0.141)
Traffic Lights × Warm Glow Net-Zero							0.400
							(0.198)
Control × Info Avoidance (PCA)							-0.0879
							(0.466)
Information × Info Avoidance (PCA)							-0.113
							(0.397)
Traffic Lights × Info Avoidance (PCA)							-0.106
							(0.420)
Control × Credibility: Carbon Neutral							0.443*
							(0.060)
Information × Credibility: Carbon Neutral							-0.261
							(0.281)
Traffic Lights × Credibility: Carbon Neutral							-0.129
							(0.593)
Control × Credibility: Net-Zero							0.317
							(0.187)
Information × Credibility: Net-Zero							0.565**
							(0.031)
Traffic Lights × Credibility: Net-Zero							-0.0132
							(0.960)
Control × Cognitive Load							0.0957
							(0.426)
Information × Cognitive Load							0.0679
							(0.577)
Traffic Lights × Cognitive Load							0.198
							(0.142)
Observations	1487	1486	1313	1102	998	990	992
Pseudo R^2	0.026	0.027	0.030	0.035	0.037	0.049	0.056
chi2	162.9	169.6	166.3	154.5	145.6	192.9	227.1

p-values in parentheses
* $p < 0.10$, ** $p < 0.05$, *** $p < 0.01$

Table 25. Determinants of overall score in the visual recognition understanding questions in Study II. Ordered logistic regression models with robust standard errors. Model 1: group assignment only. Model 2: group assignment plus basic demographics (qualification, age, gender, earnings, Democrat identification). Model 3: Model 2 plus consistency. Model 4: basic demographics, group assignment, and mechanisms (warm glow, cognitive load, information avoidance, climate change concern). Model 5: Model 4 plus consistency. Model 6: Model 5 plus credibility and interactions between the mechanisms and the treatment groups.

	(1)	(2)	(3)	(4)	(5)	(6)	(7)
	Tot U	Tot U	Tot U	Tot U	Tot U	Tot U	Tot U
Information	0.901***	0.881***	0.991***	0.955***	1.072***	1.049***	
	(0.000)	(0.000)	(0.000)	(0.000)	(0.000)	(0.000)	
Bullet Points	1.947***	1.944***	2.106***	2.054***	2.199***	2.178***	
	(0.000)	(0.000)	(0.000)	(0.000)	(0.000)	(0.000)	
Traffic Lights	1.360***	1.352***	1.404***	1.437***	1.479***	1.529***	
	(0.000)	(0.000)	(0.000)	(0.000)	(0.000)	(0.000)	
Education		-0.00471	-0.00853			-0.0207	-0.0179
		(0.799)	(0.668)			(0.360)	(0.432)
Age		-0.00408	-0.00576			-0.00761	-0.00710
		(0.219)	(0.103)			(0.102)	(0.132)
Female		0.0772	0.0368			0.00967	0.0240
		(0.473)	(0.749)			(0.942)	(0.858)
Earnings		0.0121	0.00600				
		(0.443)	(0.724)				
Democrat		0.263**	0.223*			-0.149	-0.113
		(0.015)	(0.052)			(0.393)	(0.522)
Consistency			0.309***		0.338***	0.296**	0.258*
			(0.007)		(0.010)	(0.024)	(0.051)
Warm Glow Net-Zero				0.0130	0.00718	0.0667	-0.234
				(0.904)	(0.949)	(0.555)	(0.377)
Warm Glow Carbon Neutral				-0.112	-0.139	-0.101	0.173
				(0.290)	(0.197)	(0.345)	(0.476)
Cognitive Load				-0.0951**	-0.0868**	-0.0711	-0.119
				(0.023)	(0.049)	(0.108)	(0.273)
Worry about Climate Change (PCA)				0.254***	0.230***	0.175***	
				(0.000)	(0.000)	(0.008)	
Info Avoidance (PCA)				0.0563	0.0458	0.0547	0.189*
				(0.197)	(0.322)	(0.246)	(0.064)
$10,000 to $19,999						0.178	0.236
						(0.693)	(0.601)
$20,000 to $29,999						0.560	0.557
						(0.214)	(0.207)
$30,000 to $39,999						0.218	0.223
						(0.611)	(0.595)
$40,000 to $49,999						0.236	0.391
						(0.574)	(0.344)
$50,000 to $59,999						-0.220	-0.241
						(0.608)	(0.567)
$60,000 to $69,999						0.467	0.537
						(0.302)	(0.227)
$70,000 to $79,999						0.698	0.728*
						(0.108)	(0.089)
$80,000 to $89,999						0.318	0.294
						(0.502)	(0.532)
$90,000 to $99,999						0.346	0.420
						(0.454)	(0.356)
$100,000 to $149,999						0.751*	0.804*
						(0.071)	(0.051)
$150,000 or more						0.546	0.562
						(0.215)	(0.193)
White						0.0827	0.0569
						(0.613)	(0.727)
Political Scale						-0.0791*	-0.107***
						(0.054)	(0.008)
Employed						-0.533***	-0.601***
						(0.005)	(0.001)
Retired						-0.00106	-0.112
						(0.997)	(0.682)
Republican						-0.138	-0.154
						(0.534)	(0.485)
Color Deficiency						0.00659	0.125
						(0.983)	(0.681)
Credibility: Carbon Neutral						-0.153	-0.273
						(0.121)	(0.161)
Credibility: Net-Zero						-0.0770	-0.287
						(0.384)	(0.178)
Control							-4.978***
							(0.000)
Information							-2.118*
							(0.054)
Traffic Lights							-2.303**
							(0.044)
Control × Warm Glow Carbon Neutral							-0.368
							(0.233)
Information × Warm Glow Carbon Neutral							-0.583*
							(0.073)
Traffic Lights × Warm Glow Carbon Neutral							-0.0626
							(0.837)
Control × Warm Glow Net-Zero							0.475
							(0.130)
Information × Warm Glow Net-Zero							0.563
							(0.122)
Traffic Lights × Warm Glow Net-Zero							0.390
							(0.232)
Control × Info Avoidance (PCA)							-0.142
							(0.258)
Information × Info Avoidance (PCA)							-0.151
							(0.275)
Traffic Lights × Info Avoidance (PCA)							-0.153
							(0.280)
Control × Credibility: Carbon Neutral							0.594**
							(0.021)
Information × Credibility: Carbon Neutral							-0.146
							(0.598)
Traffic Lights × Credibility: Carbon Neutral							0.0529
							(0.850)
Control × Credibility: Net-Zero							0.325
							(0.199)
Information × Credibility: Net-Zero							0.523*
							(0.060)
Traffic Lights × Credibility: Net-Zero							-0.0498
							(0.860)
Control × Cognitive Load							0.0823
							(0.515)
Information × Cognitive Load							0.0188
							(0.889)
Traffic Lights × Cognitive Load							0.160
							(0.267)
Constant	3.343***	3.326***	3.308***	4.034***	3.848***	4.858***	8.211***
	(0.000)	(0.000)	(0.000)	(0.000)	(0.000)	(0.000)	(0.000)
Observations	1487	1486	1313	1102	998	990	992
R^2	0.108	0.114	0.128	0.143	0.153	0.192	0.211

p-values in parentheses
* $p < 0.10$, ** $p < 0.05$, *** $p < 0.01$

Table 26. Determinants of overall score in the understanding questions in Study II. OLS regression models with robust standard errors. Model 1: group assignment only. Model 2: group assignment plus basic demographics (qualification, age, gender, earnings, Democrat identification). Model 3: Model 2 plus consistency. Model 4: basic demographics, group assignment, and mechanisms (warm glow, cognitive load, information avoidance, climate change concern). Model 5: Model 4 plus consistency. Model 6: Model 5 plus credibility and interactions between the mechanisms and the treatment groups.

	(1)	(2)	(3)	(4)	(5)	(6)
	Open	Open	Open	Open	Open	Open
Open						
Bullet Points	-0.0355	-0.0191	-0.0256	-0.0145	-0.00229	-0.0204
	(0.808)	(0.898)	(0.871)	(0.934)	(0.990)	(0.914)
Traffic Lights	-0.165	-0.152	-0.221	-0.150	-0.213	-0.212
	(0.252)	(0.299)	(0.155)	(0.369)	(0.227)	(0.259)
Education		-0.0222	-0.0324			-0.0652**
		(0.297)	(0.154)			(0.018)
Age		-0.0127***	-0.0131***			-0.0127**
		(0.001)	(0.001)			(0.019)
Female		-0.142	-0.157			-0.165
		(0.242)	(0.222)			(0.286)
Earnings		0.0185	-0.000419			
		(0.304)	(0.982)			
Democrat		0.301**	0.276**			0.0270
		(0.013)	(0.034)			(0.896)
Consistency			0.281**		0.265*	0.225
			(0.029)		(0.076)	(0.144)
Warm Glow Net-Zero				0.0986	0.0609	0.187
				(0.344)	(0.576)	(0.123)
Warm Glow Carbon Neutral				-0.153	-0.145	-0.0926
				(0.142)	(0.178)	(0.411)
Cognitive Load				-0.0611	-0.0601	-0.0515
				(0.205)	(0.241)	(0.332)
Worry about Climate Change (PCA)				0.121**	0.176**	0.137*
				(0.034)	(0.012)	(0.096)
Info Avoidance (PCA)				0.0974**	0.0767	0.0879*
				(0.043)	(0.130)	(0.095)
$10,000 to $19,999						0.631
						(0.233)
$20,000 to $29,999						0.569
						(0.291)
$30,000 to $39,999						0.417
						(0.408)
$40,000 to $49,999						0.454
						(0.387)
$50,000 to $59,999						0.207
						(0.687)
$60,000 to $69,999						0.866
						(0.114)
$70,000 to $79,999						0.460
						(0.388)
$80,000 to $89,999						0.153
						(0.783)
$90,000 to $99,999						0.723
						(0.201)
$100,000 to $149,999						0.801
						(0.117)
$150,000 or more						0.398
						(0.446)
White						0.166
						(0.387)
Political Scale						-0.0276
						(0.595)
Employed						-0.144
						(0.508)
Retired						-0.257
						(0.418)
Republican						-0.0958
						(0.726)
Color Deficiency						-0.660
						(0.134)
Credibility: Carbon Neutral						-0.321***
						(0.005)
Credibility: Net-Zero						-0.0159
						(0.884)
Observations	1117	1117	990	831	749	742
Pseudo R^2	0.001	0.013	0.016	0.011	0.015	0.046
chi2	1.452	22.27	24.05	14.45	17.45	53.64

p-values in parentheses
* $p < 0.10$, ** $p < 0.05$, *** $p < 0.01$

Table 27. Determinants of correct answers in the open-ended question in Study II. Ordered logistic regression models with robust standard errors excluding the control group. Model 1: group assignment only. Model 2: group assignment plus basic demographics (qualification, age, gender, earnings, Democrat identification). Model 3: Model 2 plus consistency. Model 4: basic demographics, group assignment, and mechanisms (warm glow, cognitive load, information avoidance, climate change concern). Model 5: Model 4 plus consistency. Model 6: Model 5 plus credibility and interactions between the mechanisms and the treatment groups.

	(1) Visual U	(2) Visual U	(3) Visual U	(4) Visual U	(5) Visual U	(6) Visual U
Visual U						
Bullet Points	0.896***	0.917***	0.967***	0.977***	1.017***	1.083***
	(0.000)	(0.000)	(0.000)	(0.000)	(0.000)	(0.000)
Traffic Lights	0.405***	0.415***	0.379***	0.468***	0.422***	0.531***
	(0.001)	(0.001)	(0.005)	(0.001)	(0.006)	(0.001)
Education		-0.00471	-0.00663			0.00541
		(0.793)	(0.733)			(0.819)
Age		-0.00111	-0.00260			-0.00787
		(0.741)	(0.464)			(0.127)
Female		0.0907	0.0677			0.0173
		(0.402)	(0.558)			(0.905)
Earnings		-0.0000732	-0.00433			
		(0.996)	(0.792)			
Democrat		0.202*	0.142			-0.0948
		(0.065)	(0.226)			(0.609)
Consistency			0.245**		0.269**	0.225
			(0.031)		(0.041)	(0.104)
Warm Glow Net-Zero				-0.0637	-0.0759	0.0220
				(0.583)	(0.532)	(0.859)
Warm Glow Carbon Neutral				-0.0668	-0.107	-0.102
				(0.534)	(0.323)	(0.336)
Cognitive Load				-0.0791*	-0.0759*	-0.0641
				(0.070)	(0.100)	(0.191)
Worry about Climate Change (PCA)				0.261***	0.225***	0.196***
				(0.000)	(0.000)	(0.007)
Info Avoidance (PCA)				0.0309	0.0238	0.0264
				(0.496)	(0.624)	(0.610)
$10,000 to $19,999						-0.0251
						(0.960)
$20,000 to $29,999						0.0815
						(0.873)
$30,000 to $39,999						-0.0918
						(0.845)
$40,000 to $49,999						0.103
						(0.831)
$50,000 to $59,999						-0.459
						(0.325)
$60,000 to $69,999						0.0994
						(0.842)
$70,000 to $79,999						0.541
						(0.260)
$80,000 to $89,999						0.120
						(0.820)
$90,000 to $99,999						0.0614
						(0.908)
$100,000 to $149,999						0.416
						(0.375)
$150,000 or more						0.245
						(0.616)
White						-0.0107
						(0.947)
Political Scale						-0.0890**
						(0.030)
Employed						-0.581***
						(0.002)
Retired						0.153
						(0.590)
Republican						-0.0242
						(0.919)
Color Deficiency						-0.0506
						(0.869)
Credibility: Carbon Neutral						-0.165
						(0.100)
Credibility: Net-Zero						-0.158
						(0.119)
Observations	1117	1117	990	831	749	742
Pseudo R^2	0.011	0.012	0.013	0.022	0.019	0.036
chi2	49.04	53.38	53.09	70.68	52.12	98.36

p-values in parentheses
* $p < 0.10$, ** $p < 0.05$, *** $p < 0.01$

Table 28. Determinants of overall score in the visual recognition understanding questions in Study II. Ordered logistic regression models with robust standard errors excluding the control group. Model 1: group assignment only. Model 2: group assignment plus basic demographics (qualification, age, gender, earnings, Democrat identification). Model 3: Model 2 plus consistency. Model 4: basic demographics, group assignment, and mechanisms (warm glow, cognitive load, information avoidance, climate change concern). Model 5: Model 4 plus consistency. Model 6: Model 5 plus credibility and interactions between the mechanisms and the treatment groups.

	(1)	(2)	(3)	(4)	(5)	(6)	(7)
	Tot U	Tot U	Tot U	Tot U	Tot U	Tot U	Tot U
Information	-1.047***	0	0	0	-1.125***	-0.517***	
	(0.000)	(.)	(.)	(.)	(0.000)	(0.006)	
Bullet Points	0	1.064***	1.117***	1.097***	0	0.613***	
	(.)	(0.000)	(0.000)	(0.000)	(.)	(0.002)	
Traffic Lights	-0.588***	0.470***	0.410**	0.481***	-0.714***	0	
	(0.000)	(0.003)	(0.014)	(0.006)	(0.000)	(.)	
Education		-0.0122	-0.0189			-0.0174	-0.0210
		(0.589)	(0.434)			(0.534)	(0.454)
Age		-0.00568	-0.00706			-0.0118**	-0.0115*
		(0.173)	(0.108)			(0.042)	(0.052)
Female		0.0404	0.00979			-0.0306	-0.0181
		(0.760)	(0.945)			(0.852)	(0.912)
Earnings		0.00372	-0.00472				
		(0.845)	(0.819)				
Democrat		0.318**	0.248*			-0.112	-0.144
		(0.017)	(0.082)			(0.601)	(0.506)
Consistency			0.368***		0.358**	0.264*	0.218
			(0.008)		(0.025)	(0.099)	(0.179)
Warm Glow Net-Zero				-0.0456	-0.0780	0.0349	-0.216
				(0.724)	(0.567)	(0.798)	(0.413)
Warm Glow Carbon Neutral				-0.0983	-0.139	-0.113	0.155
				(0.425)	(0.271)	(0.357)	(0.525)
Cognitive Load				-0.102*	-0.0988*	-0.0762	-0.105
				(0.054)	(0.081)	(0.179)	(0.337)
Worry about Climate Change (PCA)				0.320***	0.283***	0.221***	
				(0.000)	(0.000)	(0.007)	
Info Avoidance (PCA)				0.0577	0.0508	0.0523	0.180*
				(0.282)	(0.372)	(0.366)	(0.078)
$10,000 to $19,999						0.135	0.280
						(0.802)	(0.604)
$20,000 to $29,999						0.243	0.382
						(0.662)	(0.487)
$30,000 to $39,999						-0.0210	0.0487
						(0.968)	(0.924)
$40,000 to $49,999						0.168	0.351
						(0.749)	(0.499)
$50,000 to $59,999						-0.407	-0.376
						(0.432)	(0.461)
$60,000 to $69,999						0.309	0.508
						(0.583)	(0.359)
$70,000 to $79,999						0.680	0.761
						(0.198)	(0.144)
$80,000 to $89,999						0.134	0.182
						(0.814)	(0.747)
$90,000 to $99,999						0.291	0.420
						(0.617)	(0.468)
$100,000 to $149,999						0.708	0.761
						(0.170)	(0.138)
$150,000 or more						0.311	0.406
						(0.557)	(0.435)
White						0.0330	0.0371
						(0.866)	(0.850)
Political Scale						-0.0953*	-0.132***
						(0.054)	(0.008)
Employed						-0.692***	-0.743***
						(0.002)	(0.001)
Retired						0.102	-0.0503
						(0.755)	(0.877)
Republican						-0.103	-0.173
						(0.708)	(0.527)
Color Deficiency						-0.260	-0.149
						(0.464)	(0.669)
Credibility: Carbon Neutral						-0.278**	-0.248
						(0.020)	(0.210)
Credibility: Net-Zero						-0.135	-0.310
						(0.236)	(0.150)
Information							-2.109*
							(0.059)
Traffic Lights							-2.228*
							(0.056)
Information × Warm Glow Carbon Neutral							-0.582*
							(0.077)
Traffic Lights × Warm Glow Carbon Neutral							-0.0581
							(0.849)
Information × Warm Glow Net-Zero							0.573
							(0.120)
Traffic Lights × Warm Glow Net-Zero							0.390
							(0.227)
Information × Info Avoidance (PCA)							-0.156
							(0.261)
Traffic Lights × Info Avoidance (PCA)							-0.144
							(0.313)
Information × Credibility: Carbon Neutral							-0.171
							(0.541)
Traffic Lights × Credibility: Carbon Neutral							0.0154
							(0.957)
Information × Credibility: Net-Zero							0.544*
							(0.054)
Traffic Lights × Credibility: Net-Zero							-0.0202
							(0.943)
Information × Cognitive Load							0.0134
							(0.922)
Traffic Lights × Cognitive Load							0.147
							(0.315)
Constant	5.291***	4.381***	4.473***	5.138***	6.310***	7.460***	8.690***
	(0.000)	(0.000)	(0.000)	(0.000)	(0.000)	(0.000)	(0.000)
Observations	1117	1117	990	831	749	742	744
R^2	0.037	0.044	0.052	0.082	0.073	0.135	0.146

p-values in parentheses
* $p < 0.10$, ** $p < 0.05$, *** $p < 0.01$

Table 29. Determinants of overall score in the understanding questions in Study II. OLS regression models with robust standard errors excluding the control group. Model 1: group assignment only. Model 2: group assignment plus basic demographics (qualification, age, gender, earnings, Democrat identification). Model 3: Model 2 plus consistency. Model 4: basic demographics, group assignment, and mechanisms (warm glow, cognitive load, information avoidance, climate change concern). Model 5: Model 4 plus consistency. Model 6: Model 5 plus credibility and interactions between the mechanisms and the treatment groups.

4.3 Study III

	(1) Wrong Answers	(2) Wrong Answers	(3) Wrong Answers	(4) Wrong Answers
Wrong Answers				
Bullet Points	-0.0434	-0.0424	-0.0709	-0.0728
	(0.883)	(0.885)	(0.824)	(0.818)
Traffic Lights	0.587**	0.590**	0.516*	0.512*
	(0.027)	(0.028)	(0.067)	(0.072)
Education		-0.0330		
		(0.599)		
Age		-0.0159*	-0.0104	-0.0103
		(0.074)	(0.268)	(0.275)
Female		-0.139	-0.117	-0.119
		(0.539)	(0.628)	(0.621)
Earnings		-0.0329		
		(0.288)		
Democrat		-0.0285	-0.176	-0.177
		(0.900)	(0.545)	(0.543)
Student			0.723	0.729
			(0.160)	(0.157)
White			-0.308	-0.311
			(0.235)	(0.233)
Political Scale			-0.0613	-0.0618
			(0.479)	(0.474)
Republican			0.113	0.116
			(0.791)	(0.784)
$10,000 to $19,999			1.179	1.169
			(0.127)	(0.135)
$20,000 to $29,999			0.592	0.587
			(0.415)	(0.421)
$30,000 to $39,999			0.785	0.782
			(0.296)	(0.298)
$40,000 to $49,999			0.750	0.743
			(0.293)	(0.301)
$50,000 to $59,999			0.885	0.885
			(0.197)	(0.196)
$60,000 to $69,999			0.0517	0.0501
			(0.948)	(0.949)
$70,000 to $79,999			0.671	0.667
			(0.359)	(0.363)
$80,000 to $89,999			1.209	1.206
			(0.114)	(0.116)
$90,000 to $99,999			0.918	0.911
			(0.232)	(0.238)
$100,000 to $149,999			0.519	0.512
			(0.433)	(0.443)
$150,000 or more			-0.543	-0.551
			(0.475)	(0.472)
Some college but no degree			-0.0343	-0.0400
			(0.938)	(0.928)
College (2-year)			0.123	0.118
			(0.800)	(0.807)
College (4-year)			0.170	0.166
			(0.668)	(0.677)
Master's degree			0.163	0.163
			(0.741)	(0.740)
Professional degree (JD, MD)			0.655	0.650
			(0.428)	(0.432)
Cognitive Load				0.00980
				(0.879)
Constant	-1.486***	-0.389	-1.216	-1.323
	(0.000)	(0.465)	(0.136)	(0.211)
Observations	473	473	453	453
Pseudo R^2	0.014	0.026	0.060	0.060
chi2	7.092	12.18	30.25	30.49

p-values in parentheses
* $p < 0.10$, ** $p < 0.05$, *** $p < 0.01$

Table 30. Determinants of wrong answers in Study III. Logit regression models with robust standard errors excluding the control group. Model 1: group assignment only. Model 2: group assignment plus basic demographics (qualification, age, gender, earnings, Democrat identification). Model 3: all demographics, group assignment. Model 4 all demographics and cognitive load.

5 T-tests: Robustness checks for Understanding

5.0.1 Understanding

As a robustness check, we replicated the analysis using our own coding of the open-ended question in Study II. Overall, we found that the results are consistent with the ones presented in the article. We first studied the differences within and across groups through t-tests. For within-group comparisons p-values and Cohen's d are computed using a two-sided paired t-test with unequal variances, while for within-target comparisons we reported the results of two-sample two-sided t-test using unequal variances. We continued to observe that all treatments significantly improved respondents' total understanding with respect to the control group.

	Control	Information	Bullet points	Traffic lights
Open question	0.086	0.253	0.334	0.222
Difference (total und. v. control)	-	0.167*** (0.031)	0.247*** (0.032)	0.135*** (0.028)
p	-	<0.001	<0.001	<0.001
Cohen's d	-	0.402	0.5614	0.359

Table 31. Understanding differences across the treatment groups with the stricter criteria for the open-ended questions in Study II. The p-values reported are all for a two-sided paired t-test with unequal variances. Standard errors are reported in parentheses.

In line with the main body, to further illustrate the impact of the treatments we reported the odds ratio from a regression controlling for information avoidance, cognitive overload, credibility, warm glow, consistency and demographics in table 32. The impact of bullet points and information remained highly significant, while that of traffic lights disappeared.

	Group		
	Bullet Points	Information	Traffic lights
Open Question	98.51 (p=0.001)	24.74 (p= 0.038)	8.26 (p=0.148)

Table 32. Odds ratios and p-values (in parentheses) from an ordered logit regression interacting the treatment groups with information avoidance, information overload, credibility, warm glow, consistency and controlling for participants' demographics (Study II).

We also observed significant differences among treatments. Respondents in the bullet points condition outperformed both respondents in the information condition ($p = 0.0413$, Cohen's d=0.15) and in the traffic lights condition ($p = 0.003$, Cohen's d=0.22), while respondents in the traffic lights condition did worse than those in the information condition, but this result was not significant ($p = 0.38$, Cohen's d=0.06).

References

1. Dunn, M. J. *et al.* Minimal reporting guideline for research involving eye tracking (2023 edition). *Behavior research methods* **56**, 4351–4357 (2024).
2. Holmqvist, K. *et al. Eye tracking: A comprehensive guide to methods and measures* (oup Oxford, 2011).
3. Hooge, I. T. *et al.* The fundamentals of eye tracking part 2: From research question to operationalization. *Behavior Research Methods* **57**, 73 (2025).
4. Fuhl, W., Kübler, T. C., Santini, T. & Kasneci, E. Automatic generation of saliency-based areas of interest for the visualization and analysis of eye-tracking data. In *VMV*, 47–54 (2018).
5. Orquin, J. L., Ashby, N. J. & Clarke, A. D. Areas of interest as a signal detection problem in behavioral eye-tracking research. *Journal of Behavioral Decision Making* **29**, 103–115 (2016).
6. Hessels, R. S., Kemner, C., van den Boomen, C. & Hooge, I. T. The area-of-interest problem in eyetracking research: A noise-robust solution for face and sparse stimuli. *Behavior research methods* **48**, 1694–1712 (2016).
7. Vehlen, A., Standard, W. & Domes, G. How to choose the size of facial areas of interest in interactive eye tracking. *PLoS One* **17**, e0263594 (2022).
